# Eradication of unresectable liver metastasis through induction of tumour specific energy depletion

**DOI:** 10.1038/s41467-019-11082-3

**Published:** 2019-07-11

**Authors:** Da Huo, Jianfeng Zhu, Guojun Chen, Qian Chen, Chao Zhang, Xingyu Luo, Wei Jiang, Xiqun Jiang, Zhen Gu, Yong Hu

**Affiliations:** 1 Collaborative Innovation Center of Chemistry for Life Sciences, College of Engineering and Applied Sciences, Nanjing University, Nanjing, Jiangsu China; 2 Department of Bioengineering and the California Nanosystems Institute, University of California, Los Angeles, CA USA; 3 Jonsson Comprehensive Cancer Center and Center for Minimally Invasive Therapeutics, University of California, Los Angeles, CA USA; 4 Department of Polymer Science & Engineering, College of Chemistry & Chemical Engineering, Nanjing University, Nanjing, Jiangsu China

**Keywords:** Cancer, Materials science, Nanoscience and technology

## Abstract

Treatment of liver metastasis experiences slow progress owing to the severe side effects. In this study, we demonstrate a strategy capable of eliminating metastatic cancer cells in a selective manner. Nucleus-targeting W_18_O_49_ nanoparticles (WONPs) are conjugated to mitochondria-selective mesoporous silica nanoparticles (MSNs) containing photosensitizer (Ce6) through a Cathepsin B-cleavable peptide. In hepatocytes, upon the laser irradiation, the generated singlet oxygen species are consumed by WONPs, in turn leading to the loss of their photothermally heating capacity, thereby sparing hepatocyte from thermal damage induced by the laser illumination. By contrast, in cancer cells, the cleaved peptide linker allows WONPs and MSNs to respectively target nucleus and mitochondria, where the therapeutic powers could be unleashed, both photodynamically and photothermally. This ensures the energy production of cancer cells can be abolished. We further assess the underlying molecular mechanism at both gene and protein levels to better understand the therapeutic outcome.

## Abstract (web summary)

Treatment of liver metastasis in cancer patients is associated with severe side effects. Here, the authors develop nucleus and mitochondria targeted nanoparticles, conjugated via a cathepsin B sensitive peptide to selectively target liver metastatic cells.

## Introduction

Among various organs, liver is the most common site for colonization of cancer cells, resulting in a high incidence of liver metastasis and a high risk of cancer-related death^1–3^. So far, surgery is the most effective approach in handling liver metastasis in the clinic^2^. Unfortunately, most cases of liver metastases are diagnosed at the late stage when surgical means is no longer effective. The case grows even worse for diffusive liver metastasis (DLM) as characterized by the presence of multiple metastasis nodules^4^. As an organ responsible for intense metabolic activities, the liver itself is highly vulnerable to complications associated with chemotherapeutics, thereby dampening the survival benefits brought by chemotherapy^5,6^. Some of the emerging therapeutics, such as iniparib and niraparib, are capable of attenuating only cancer cells carrying unique genomic feature by harnessing the mechanism named synthetic lethality^7^. Though helpful to some extent, no success has been witnessed in the case of liver metastasis yet. Besides, the precision down to molecule level can, in turn, become a drawback for lacking a potential of general application^8^. As such, it is still an urgent task to develop an effective approach to extend the lifespan of DLM-suffering patients.

By virtue of the fact that nanomaterials could favorably accumulate in the liver after their blood entry^8–13^, nanomedicine is now growingly recognized as a promising weapon against liver abnormities^14–16^. The introduction of targeting moieties was expected to promote nanomaterials’ enrichment in diseased sites^17–22^. However, targeting specific biomarkers only allows a certain portion of patients to benefit from the therapeutic^23^. To manage pathologically distinct cancers, approach targeting more universal features should be developed. Here, we focus on the energy machinery of cancer cells^24,25^. On top of biogenesis stands the ATP (adenosine triphosphate) as the fuel. Initial attempts of blocking glycolysis (GLY)-related pathways were made given the metabolic preference of cancer cells toward GLY over oxidative phosphorylation (OXPHOS). Such a phenomenon is referred to as Warburg effect^26^. Now, it is clear that some intermediates produced during GLY are behind the metabolic shift to GLY^26–29^. The OXPHOS can undergo a compensatory activation once GLY is inhibited in an effort to rescue the stressed cancer cells^26^. To conquer a tumour, concurrent inhibition of OXPHOS and GLY activities is necessary. Meanwhile, as normal cells also rely on OXPHOS to support their homeostasis, it is practically challenging to confine the action only to cancer cells.

**Fig. 1:**
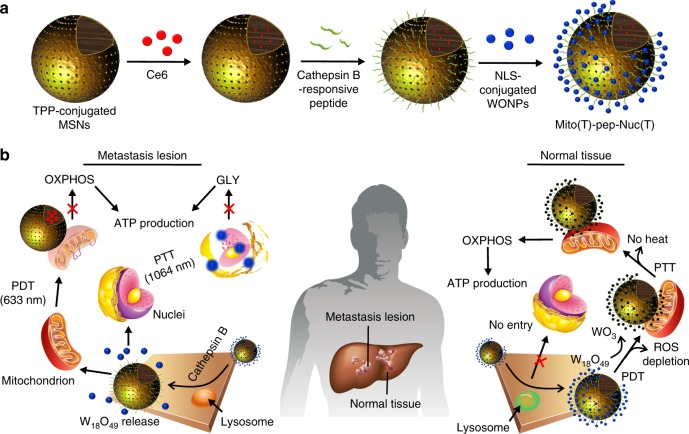
Working principle of Mito(T)-pep-Nuc(T). **a** Synthetic procedure of the mitochondria and nucleus dual-targeting platform, Mito(T)-pep-Nuc(T). **b** A schematic illustration showing the mechanism that synthesized nanomedicine could distinguish metastasis lesions from normal liver parenchyma. In cancer cells metastasized to the liver, the internalized Mito(T)-pep-Nuc(T) will be destabilized upon the cleavage mediated by the overexpressed Cathepsin B enzyme. The released MSNs and W_18_O_49_ NPs will then respectively target mitochondria and nucleus, consequently leading to their impairment upon laser irradiation (633 nm followed by 1064 nm in order to trigger PDT and PTT, respectively). In hepatocytes, the internalized Mito(T)-pep-Nuc(T) remains morphologically intact. Upon the irradiation with 633-nm laser, the consumption of singlet oxygen by WONPs could result in their oxidation into WO_3_ featuring attenuated heating power upon subsequent laser irradiation (1064 nm). On this basis, hepatocytes and metastasis cancer cells are being treated differently, leaving only the latter one be ablated. TPP triphenyl phosphonium, WONPs W_18_O_49_ nanoparticles, OXPHOS oxidative phosphorylation, GLY glycolysis, PDT, photodynamic therapy, PTT photothermal therapy

In this study, we proposed to selectively eliminate metastatic cancer cells through energy depletion. A hybrid system composed of photosensitizer (Ce6)-encapsulated mesoporous silica nanoparticle (MSN) and W_18_O_49_ nanoparticles (WONPs) was synthesized. As shown in Fig. 1a, the MSNs and WONPs were functionalized with triphenyl phosphonium (TPP) and nuclear localization sequence (NLS), respectively, which allow their selective binding with mitochondria^30^ and nucleus^31^. The nucleus-targeting WONPs (Nuc(T)) were next covalently conjugated to mitochondria-targeting MSNs (Mito(T)) via a peptide cleavable by Cathepsin B (final product denoted by Mito(T)-pep-Nuc(T)), whose expression is aberrantly high in a variety of cancers^32^. As illustrated in Fig. 1b, in cancer cells, Mito(T)-pep-Nuc(T) is expected to rupture in the Cathepsin B-rich lysosomes, liberating Mito(T) and Nuc(T) with distinct subcellular destinations. Afterward, the laser irradiations are applied (633 nm followed by 1064 nm) to sequentially trigger the photodynamic therapy (PDT) and photothermal therapy (PTT) mediated by Ce6 and WONPs, respectively. Both organelles could be destroyed, collectively abolishing the ATP supply. Meanwhile, the peptide linker stays intact in hepatocytes due to the insufficient Cathepsin B activity. Upon 633-nm laser irradiation, the generated singlet oxygen could be stoichiometrically consumed by WONPs, thereby turning them into photothermally inert WO_3_ and abolishing the heat generation upon 1064-nm laser exposure. As such, the hepatocytes could be rescued from the destruction caused by both PDT and PTT.

## Results

### Synthesis of the platform and the optimization

The MSNs were synthesized following a published protocol^33^ featuring homogeneous pores with a mean diameter of 2.3 nm (Supplementary Fig. 1). The WONPs were synthesized according to our previous publication^34,35^, followed by their conjugation onto MSN that contributes to a planet–satellite-like structure (Supplementary Fig. 2). Enlargement in size was confirmed by dynamic light scattering (DLS, Supplementary Fig. 3) analysis. The mean Zeta-potential (Supplementary Fig. 4) decreased slightly from 36.7 to 28.7 mV for APTES- and peptide-functionalized MSNs, respectively, and drastically decreased to −3.9 mV post WONPs conjugation, possibly owing to the presence of carboxyl group in WONPs. Afterward, we analyzed the enzymatic response of Mito(T)-pep-Nuc(T). The resultant nanoparticles were incubated with several enzymes and size changes were recorded by DLS analysis (Supplementary Fig. 5). A noticeable size reduction can only be observed in the presence of Cathepsin B (from 138.7 to 99.1 nm) owing to the detachment of WONPs. This is further supported by the TEM results (Supplementary Fig. 6), wherein the released WONPs could be found. The content of tungsten in Mito(T)-pep-Nuc(T) was assessed using inductively coupled plasma-mass spectra (ICP-MS) before and after the enzyme challenge (Supplementary Fig. 7). Exposure to Cathepsin B resulted in the release of 93.7% of the anchored WONPs, whereas only 2.0% liberation was found in the presence of Caspase 3 and MMP-2 (Supplementary Fig. 8).

**Fig. 2:**
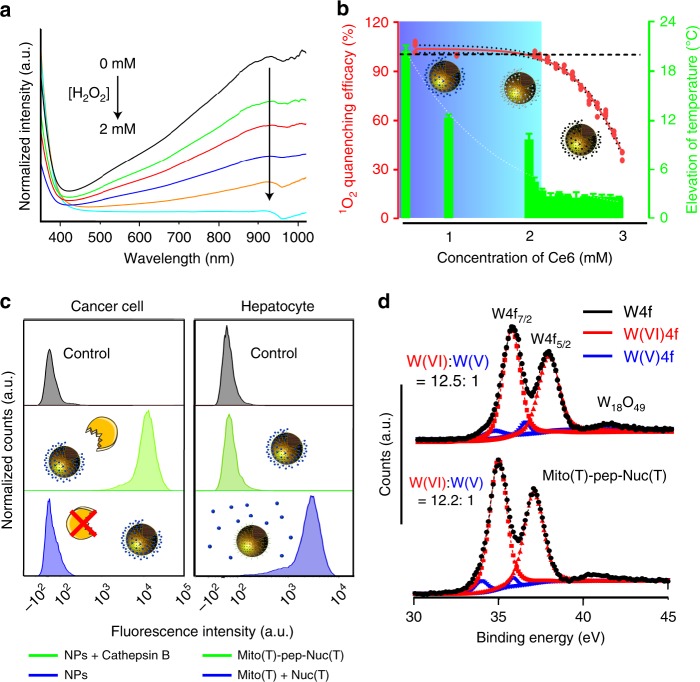
Optimization of the composition. **a** UV–vis–NIR spectra of the WONPs suspension post the addition of different amounts of H_2_O_2_. The oxidation of WONPs into WO_3_ was reflected in real-time by the loss of the characteristic peak at 930 nm. **b** The efficacy of WONPs at a fixed concentration (2.4 × 10^16^ particles per mL) in eliminating singlet oxygen generated from Ce6 that differed in amount. The temperature elevation of the suspension was measured right after the second-round laser irradiation. Data are presented as mean ± s.e.m. (*n* = 3). The cartoons inserted in the image illustrate the changes of WONPs that were partially (middle) to entirely oxidized (right). **c** The level of intracellular ROS measured using the H_2_DCFDA probe. For cancer cells, they were all incubated with Mito(T)-pep-Nuc(T), and the only difference is the enzymatic activity of Cathepsin B (normal: NPs + Cathepsin B; inhibited: NPs). For hepatocytes, they were incubated with either Mito(T)-pep-Nuc(T) or a mixture of Mito(T) and Nuc(T) under an identical condition. **d** The XPS spectra of tungsten element in Nuc(T) of Mito(T)-pep-Nuc(T) at Day 4 post their incubation with mouse serum at 37 °C. The plot was deconvoluted to show the proportions of both W(V) and W(VI) species. The profiles of pristine WONPs served as control and was shown for comparison

Next, we optimized the formulation of Mito(T)-pep-Nuc(T). In principle, the generated singlet oxygen species (SOS) should be stoichiometrically consumed by WONPs. We used H_2_O_2_ as a mimic of SOS. Ce6-free Mito(T)-pep-Nuc(T) was incubated with different amounts of H_2_O_2_. We noticed that the introduction of H_2_O_2_ proportionally reduced the intensity of absorption of WONPs at 930 nm (Fig. 2a). Typically, the number of WONPs that can be conjugated to an individual MSN was measured to be around 235, giving a total concentration of 2.4 × 10^16^ WONPs per mL solution. We next fixed the concentration of WONPs, and adjusted the amount of Ce6 payload to the one best fulfilling the requirements of the SOS depletion and heating power abolishment. As calculated, those WONPs (~4.5 nm in diameter) are expected to consume 1.5-fold more oxygen species, that is, 5.3 μmol SOS. Under irradiation duration (15 min, 1 W cm^−2^), given a photon generation efficacy of 3.7 × 10^−3^ cm^2 ^mW^−1^ s^−1^, a 63.0% quantum yield, and an extinction coefficient as 3.5 × 10^−3^ (ref. ^36^), the optimal concentration of Ce6 was calculated to be 1.7 mM. As such, we prepared various platforms carrying different amounts of Ce6. Both the SOS levels in Mito(T)-pep-Nuc(T) and control (Ce6 alone) groups were measured post illumination. We further calculated the extent of SOS consumption. We also recorded the temperature profiles after the second-round irradiation (1064-nm laser; Fig. 2b). Not surprisingly, a reduced WONPs availability led to weakened heating power. Moreover, we noticed that the optimal concentration of Ce6 herein was determined to be 2.1 mM, slightly higher than the predicted value, presumably, due to heterogeneous illumination payloads received that differed from the condition used for theoretical calculation. The UV–vis–NIR spectra of optimized samples before and after 633-nm laser irradiation were also recorded (Supplementary Fig. 9). As expected, the absorption of WONPs in NIR region decreased accordingly. Besides, we found the irradiation with 633-nm laser caused minor temperature elevation (<2.5 °C, Supplementary Fig. 10), possibly owing to the ever-decreasing optical absorption of WONPs. The release profiles of Ce6 with or without Cathepsin B enzymes were also evaluated as shown in Supplementary Fig. 11. With respect to the slow premature release of Ce6, the participation of enzyme noticeably promoted the liberation (~10.0% within 8 h). We believe that both the electrostatic interaction between Ce6 and amine-functionalized MSNs and the capping effect of WONPs help inhibit the premature release.

### Cellular uptake and distribution

Before moving to in vivo analysis, we assessed the performance of our platform in vitro. As shown in Fig. 2c, the intracellular levels of ROS in both HCT-116 (human primary colon cancer cell line) and HHL-5 (hepatocyte cell line) were relatively low. Upon the laser irradiation, it substantially increased by 182-fold in HCT-116 cells that received Mito(T)-pep-Nuc(T) (Supplementary Fig. 12), whereas hepatocytes were negligibly influenced. Once the activity of Cathepsin B was inhibited, the ROS generation was attenuated in cancer cells. Furthermore, the HHL-5 cells, when incubated with a mixture of Mito(T) and Nuc(T), cannot be rescued as effectively as did by Mito(T)-pep-Nuc(T) (Supplementary Fig. 12). One may argue that it was the reduced internalization of Nuc(T) which was behind the inadequate scavenging. We thus analyzed the content of tungsten of cells incubated with either Mito(T)-pep-Nuc(T) or Nuc(T) (2.4 × 10^16^ WONPs per mL; Supplementary Fig. 13). Less uptake of Nuc(T) was noticed, accounting for 87.2% of that of Mito(T)-pep-Nuc(T). As such, one can still expect the vast of generated SOS to be depleted. The distance between Nuc(T) (in the nucleus) and SOS (nearby mitochondria) might be part of the reasons why the scavenging capacity of Nuc(T) was weakened. Furthermore, we assessed the serum stability of Mito(T)-pep-Nuc(T). Figure 2d shows the X-ray photoelectron spectra (XPS) results of WONPs before and after incubation. Clearly, the WONPs stayed intact as reflected by the almost constant W(V) to W(VI) ratio, thus ruling out the possibility of WONPs undergoing premature oxidation.

**Fig. 3:**
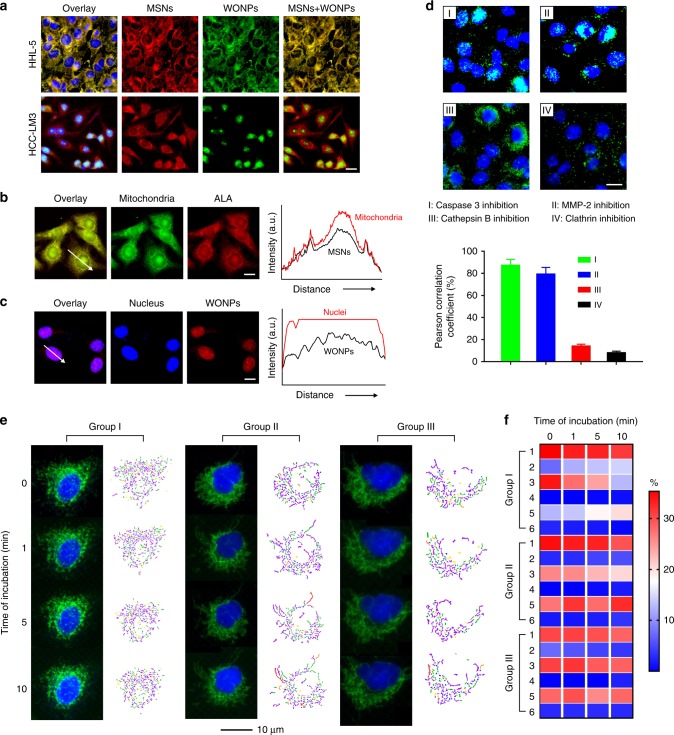
In vitro cellular uptake and targeting. **a** The fluorescence micrographs of HHL-5 (hepatocyte) and HCT-116 (cancer cell) cells post incubation with Mito(T)-pep-Nuc(T)(Alex Fluor-488-labeled) for 4 h. The nuclei of the cells were stained with Hoechst after the incubation with nanoparticles. Scale bar: 10 μm. **b**, **c** Correlation of **b** Mito(T)-pep-Nuc(T)(no labeling) with mitochondria (stained with Mitotracker Green) and **c** Mito(T)(no payload)-pep-Nuc(T)(dye-labeled) and nuclei (stained with Hoechst) post a 4 h incubation with HCT-116 cells. Their fluorescence intensity profiles were measured using Fiji (Image J) and shown as a function of distance on the right. **d** the correlation of dye-labeled Nuc(T) with nuclei post a 4 h incubation of Mito(T)-pep-Nuc(T) with HCT-116 cells whose activity of Caspase 3, MMP-2, Cathepsin B, or Clathrin was blocked. Scale bar: 10 μm. The Pearson correlation analyses of the fluorescence micrographs were shown at the bottom (data are presented as mean ± s.e.m., *n* = 4). **e** In situ time-lapse fluorescence study of the changes in mitochondrial morphology during laser irradiation (633 nm, 1 W cm^−2^). The HCT-116 cells were pre-incubated with Mito(T)-pep-Nuc(T). The observation was conducted in triplicate as denoted by Group I–III, with the microstructures of mitochondria analyzed using MicroP software, including six classes of mitochondria as detailed in the captions (I–VI). **f** The populations of six phenotype mitochondria as a function of the duration of laser irradiation. A color bar on the right indicates the color scales. *P* values were calculated using one-Way ANOVA (***p < 0.001)

Furthermore, the subcellular distribution of Mito(T)-pep-Nuc(T) post endocytosis was analyzed. For Nuc(T), they were labeled with Alexa Fluor-488 in order to make them distinguishable from Ce6-loaded Mito(T). Figure 3a shows the fluorescence micrographs of cells after the incubation with nanoparticles for 4 h. In hepatocytes, the Ce6 fluorescence of Mito(T) overlapped with that of Nuc(T), indicating no sign of structure rupture. By contrast, in HCT-116 cells, the pattern of Mito(T) nanoparticles differed considerably from that of Nuc(T). To precisely analyze their distribution, mitochondria and nucleus were stained individually post the incubation with Mito(T)(no payload)-pep-Nuc(T)(dye-labeled) and Mito(T)(Ce6-loaded)-pep-Nuc(T)(no label), respectively. We confirmed the accumulation of Mito(T) and Nuc(T) in mitochondria and nucleus (Fig. 3b, c), respectively. Furthermore, we have observed the time-dependent enrichment of WONPs in the nucleus (Supplementary Fig. 14). Specifically, diffusive distribution of the internalized WONPs after a 30-min incubation indicated the escape of Nuc(T) from endolysosomes^37^. The overlapping between Hoechst and Alexa Fluor-488 indicated the nuclear entry of Nuc(T), which was accomplished within 90 min. When we inhibited the activities of several enzymes before the incubation with Mito(T)-pep-Nuc(T)(dye-labeled), we found that only the impaired Cathepsin B blocked nuclear-entry of Nuc(T) (Fig. 3d). We also proved that the internalization of Mito(T)-pep-Nuc(T) was Clathrin-dependent on the basis of fact that the presence of Clathrin inhibitor, chlorpromazine (Fig. 3d)^38^, noticeably decreased the endocytosis.

### Morphology changes of mitochondria

An in situ observation was conducted using fluorescence imaging equipped with a live cell culture chamber. Figure 3e shows three representative cells, whose fluorescence micrographs were recorded in parallel during the laser irradiation. Based on these results, the populations of six classes of mitochondria were analyzed and displayed on the right panel of each group^39,40^. The population of each subtype as a function of irradiation duration was summarized in a heat-map (Fig. 3f). We found that the small globule-like mitochondrion was least prone to PDT, whereas the branched tubule-like and twisted counterparts exhibited the highest vulnerability. In Groups I and II, the population of branched tubule-like mitochondria reduced over time, in directly opposite to that of those twisted counterparts. The measured mean aspect ratio of mitochondria also saw a decrease over time in all three groups (Supplementary Fig. 15). It coincided with the increasing population of the small globule, whose aspect ratio was the smallest among all^39^. Considering the heterogeneity of mitochondria, our results suggested that subtype greater in length might be more vulnerable to PDT than its shorter counterparts do.

### Activities of OXPHOS and GLY

**Fig. 4:**
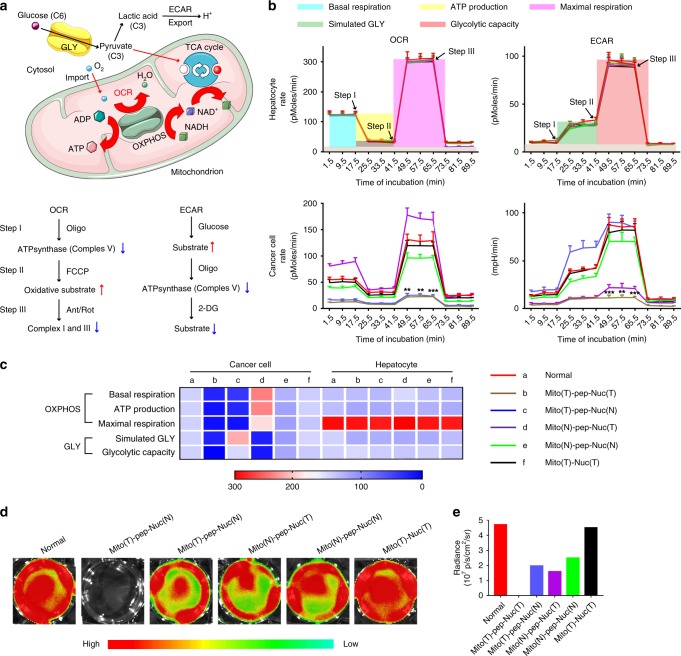
GLY and OXPHOS activities of treated cells. **a** A schematic illustrating how GLY and OXPHOS are executed, along with the network showing the essential species involved in these two processes. The chemical effectors involved during the measurement of OCR and ECAR are shown at the bottom. Red and blue arrows stand for the up- and down-regulation of metabolic activities, respectively. **b** Profiles of OCR and ECAR measured by a Seahorse XF-24 analyzer, whose level reflected the activities of OXPHOS and GLY in real time. Such an analysis was conducted right after the two-round laser irradiation. Data are presented as mean ± s.e.m. (*n* = 3). **c** A heat-map showing the values of basal respiration, ATP production, maximal respiration, and the simulated GLY and glycolytic capacity that were respectively associated with OXPHOS and GLY. These profiles were measured on the basis of plots shown in **b**. The captions shown on the right are valid for both **b** and **c**. A color bar on the right indicates the color scales. **d** Bioluminescence micrographs of luciferase-transfected HCT-116 cells that were incubated with different types of platforms before laser irradiation. d-Luciferin was added into the culture medium of cells right after the laser irradiation to show the availability of ATP. **e** Radiance intensity of cells shown in **d**. The “T” and “N” were used to demonstrate the presence and absence of targeting moieties toward either nucleus or mitochondrion, respectively. For the Mito(T)-Nuc(T), it represented the platform composed of nuclear-targeting WONPs (Nuc(T)) and mitochondrion-targeting MSNs (Mito(T)) that was covalently linked to each other via a linker that was non-cleavable by Cathepsin B. *P* values were calculated by one-way ANOVA (***p < 0.001, **p < 0.005)

We used the Seahorse XF Extracellular Flux analyzer to record the values of oxygen consumption rate (OCR) and the extracellular acidification rate (ECAR)^40^. Figure 4a illustrates a typical metabolic process that the glucose could undergo in cancer cells. First, the glucose is catabolized to pyruvate, an intermediate fueling both OXPHOS and GLY^26^. During OXPHOS, the pyruvate enters the tricarboxylic acid (TCA) cycle along with oxygen, thereby makes it feasible to monitor the activity of OXPHOS by measuring the OCR^40^. Alternatively, the pyruvate can be engineered into lactate (in GLY), the exocytosis of which acidified the extracellular milieu as measured by the changes in ECAR^40^. Here, platforms differed in their organelle selectivity were eveluated (Fig. 4b and Supplementary Fig. 16). Analyses of both OCR and ECAR can be divided into three stages involved in the step-wise introduction of chemical effectors (Fig. 4a). During OXPHOS, the OCR at the basal level is measured before the ATP synthase (Complex V) is inhibited by Oligomycin^40^. Next, the mitochondrial membrane is permeabilized with FCCP (carbonyl cyanide-4 (trifluoromethoxy) phenylhydrazone), constantly maximizing the OCR due to unregulated electron flow^40^. Both the upper limit of OXPHOS and the spare respiratory capacity can be measured simultaneously. Afterward, a mixture of rotenone and antimycin A was introduced that respectively inhibit the functions of Complex I and III^40^, collectively blocking OXPHOS. For the ECAR measurement, glucose, Oligomycin, and 2-DG (2-deoxy-glucose) are sequentially added during steps I–III, in order to provide substrates for GLY, inhibit the Complex V, and abrogate GLY through competitive binding with glucose hexokinase^40^, the upper-stream enzyme of the glycolytic pathway. Specifically, as shown in Fig. 4b, it was found that the treatments mediated by all these five platforms, including the Mito(T)-pep-Nuc(T), Mito(T)-pep-Nuc(N), Mito(N)-pep-Nuc(T), Mito(N)-pep-Nuc(N), and Mito(T)-Nuc(T), possess negligible threat to hepatocytes as reflected by their OXPHOS activities. The calculated values of basal respiration rate, ATP production, and the maximal respiration capacity, as displayed in a heat-map (Fig. 4c) supported this assertion. Likewise, the ECAR profiles of hepatocytes indicated GLY activity was also minorly influenced (Fig. 4b, c). Our previous findings suggested that platforms of this kind could keep intact after its internalization into cells. As such, the expected reaction between SOS and WONPs as well as the loss of heating power of the latter one can take place at wherever the whole platform accumulated. Conversely, individual components like Mito(T/N) and Nuc(T/N) were not suitable for future tests owing to their negative influence on either OCR (Supplementary Fig. 16) or ECAR (Supplementary Fig. 17) of hepatocytes. Due to the safety concern, only the five hybrid platforms were discussed and exploited further.

In cancer cells, these platforms affected metabolic activities differently. The treatment mediated by Mito(T)-pep-Nuc(T) resulted in the strongest inhibition in activities of both OXPHOS and GLY, whereas Mito(T)-pep-Nuc(N) and Mito(N)-pep-Nuc(T) only shut down the energy produced through OXPHOS and GLY, respectively. Of note, the OCR activity of cells received Mito(N)-pep-Nuc(T) was increased to a level higher than that for normal cancer cells, revealing that OXPHOS could compensate the energy loss due to GLY blockage, coinciding that described in literature^26^. Although the safety of both Mito(N)-pep-Nuc(N) and Mito(T)-Nuc(T) was assured, their therapeutic performances in vitro were unsatisfactory; the activity of neither type of metabolism experienced a notable reduction, making the in vivo exploration of less value. Using the luciferase-based assay, we also quantified the level of intracellular ATP. Figure 4d shows the bioluminescence graphs of cells received different treatments. It is found that the ATP in cells treated with Mito(T)-pep-Nuc(T) was reduced to a level below the detection limit of bioluminescence imaging (BLI) modality (Fig. 4e), whereas the Mito(T)-Nuc(T) led to the minimal reduction. Besides, the ATP level in cancer cells treated with Mito(T)-pep-Nuc(N) was higher than that of Mito(N)-pep-Nuc(T) group (Fig. 4e), suggesting a stronger capacity of GLY in compensating the energy loss relative to OXPHOS. Possibly, it can be explained by the faster action of GLY relative to OXPHOS.

### Pharmacokinetic and apoptotic profiles

**Fig. 5:**
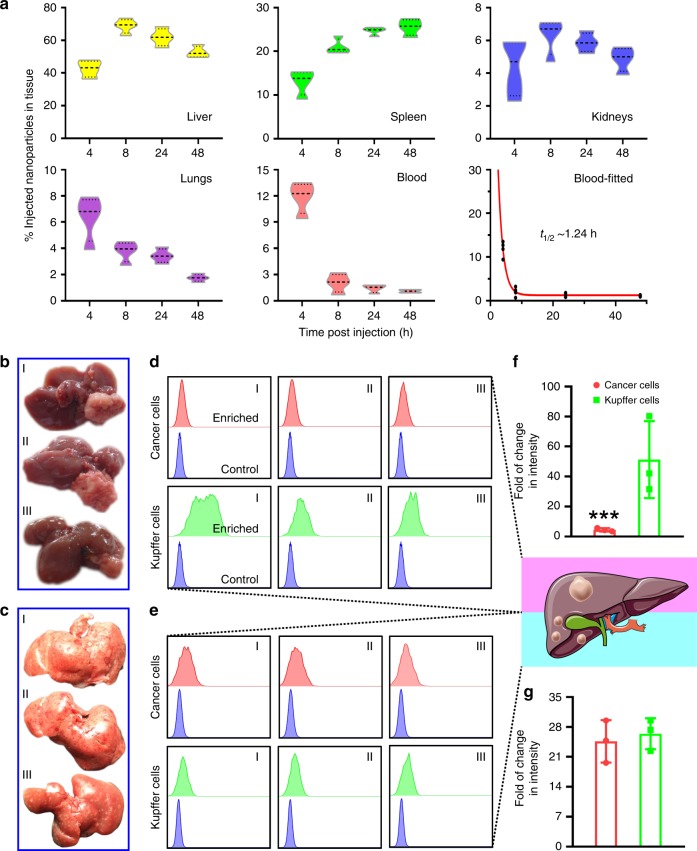
Pharmacokinetic analysis. **a** The bio-distribution profiles of Mito(T)-pep-Nuc(T). Mice bearing DLM were injected intravenously with Mito(T)-pep-Nuc(T) before sacrificed at different time points. Four organs including liver, spleen, kidneys, and lungs were collected in addition to blood sample. The violin plot outlines illustrate kernel probablity density, with the area revealing the population of located data. Data are presented as mean ± s.e.m. (*n* = 3). **b**, **c** Necropsy images of liver tissues bearing **b** HCC and **c** DLM. These tissues were sampled as I to III, followed by processing and analyses individually. **d**, **e** shows the flow-cytometry results of cancer cells and Kupffer cells enriched from the single cell suspension produced from **d** HCC and **e** DLM. In each graph, the upper row displays the histogram of enriched cells, with the lower row lays the same control cells received no treatment. A cartoon was given right to **d** and **e**, wherein the simplified pathological features of HCC and DLM were shown. **f**, **g** The averaged results of fluorescence intensity change (Ce6) in a fold of change fashion for **f** HCC and **g** DLM model, respectively. Data are presented as mean ± s.e.m. (*n* = 3). *P* values were calculated by Student’s *t-*test (***p < 0.005)

The green fluorescence protein (GFP)/luciferase-transfected HCT-116 cells (HCT-116^*GFP/Luc*^) were used for establishing DLM animal model. Next, we evaluated the bio-distribution profiles of Mito(T)-pep-Nuc(T). Figure 5a shows the number of nanoparticles in different tissues as a function of time. Maximal accumulation of Mito(T)-pep-Nuc(T) in the liver was witnessed at 8 h (68.8% of the total injection), followed by slow clearance. The blood circulation profile was also assessed, with the half-life of nanoparticle estimated to be 1.24 h. Here, the declined blood availability agreed well with the quick entry of nanoparticles into the reticuloendothelial system (liver and spleen). Such a manner effectively helps reduce the duration of time necessitated for injected nanoparticles to enrich in the liver to a noticeable extent.

We next managed to understand the distribution profiles of Mito(T)-pep-Nuc(T) at the cell level. Specifically, mice bearing primary hepatocellular carcinoma (HCC) or DLM were involved. In both models, they received a tail vein injection of Mito(T)-pep-Nuc(T), with an interval of 8 h allowed for these injected nanoparticles to accumulate in the liver. Figure 5b, c shows the necropsy images of liver tissues collected from HCC- and DLM-bearing mice, respectively. They were then processed into single-cell suspension, with both cancer and Kupffer cells sorted according to the expression of GFP and F4/80, respectively. Intracellular Ce6 fluorescence was recorded using flow-cytometry (Fig. 5d, e for HCC and DLM, respectively), with the changes in intensity calculated by normalizing the measured value to that of control cells received no treatments (Fig. 5f, g for HCC and DLM, respectively). The distribution of nanoparticles in the presence of HCC meets our expectation, wherein the enrichments in Kupffer cells outnumbered that in cancerous counterparts. This can be, at least partially, explained by the lack of deep tumour penetration capacity of nanoparticles against the high interstitial pressure. By contrast, the much smaller volumes of metastasis nodule (around 1/10–1/5 size of the primary tumour) as observed in DLM allows cancer cells to compete more strongly with Kupffer cells for nanoparticles. This was supported by the observation that accumulation of nanoparticles in Kupffer cells was only slightly higher than in cancer cells (Fig. 5g).

Furthermore, we assessed the therapeutic potential of Mito(T)-pep-Nuc(T). Sorafenib, the first-line therapeutic for liver cancer^41^, was also included. All mice other than those in sorafenib and control groups received laser treatment at 24 h post the administration of therapeutics. We confirmed that the exposure to 633-nm laser was safe in our case. Supplementary Figure 18 shows the changes in three biomarkers, including alanine aminotransferase (ALT), total protein (TP), and total bilirubin (TBIL). The expression levels of TP and TBIL varied shortly after laser exposure and recovered within 2 weeks. This reveals that the stress condition induced by illumination was temporal and overall tolerable.

**Fig. 6:**
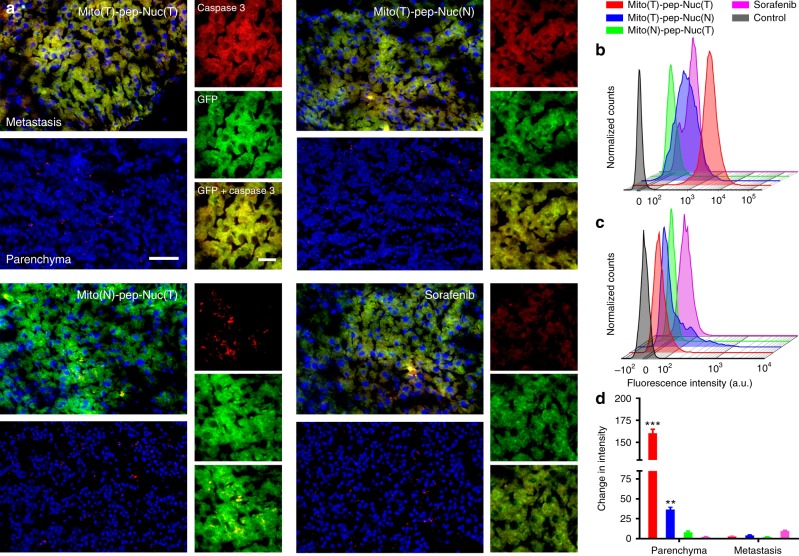
Apoptosis profiles of liver metastatic lesion and parenchyma. **a** Immunofluorescence micrographs showing the expression of active Caspase 3 (labeled in red) in both metastasis (GFP positive, green) and pan-cancer, normal parenchyma (GFP negative). Scale bars: 80 μm (left) and 20 μm (right). **b**, **c** Flow-cytometry results showing the intracellular cleaved-PARP expression. The freshly harvested liver tissues from mice received different treatments were digested and sorted using flow-cytometry on the basis of the expression of the GFP protein, sequentially followed by fixation, permeabilization, and staining with phycoerythrin (PE)-labeled cleaved-PARP antibody. **d** Changes of cleaved-PARP expression in **b** metastasis and **c** liver parenchyma. The averaged value of three independent tests was normalized to that of the control group; data are presented as mean ± s.e.m. (*n* = 3). *P* values were calculated by one-way ANOVA (***p < 0.001, **p < 0.005)

We next conducted sequential laser irradiation. Figure 6a shows the representative immunofluorescence micrographs of liver tissues with active Caspase 3, a biomarker of apoptosis^42^. The metastasis lesions can be readily recognized based on their GFP expression. Massive cell death in metastasis was witnessed in mice received treatments mediated by either Mito(T)-pep-Nuc(T) or Mito(T)-pep-Nuc(N), but not Mito(N)-pep-Nuc(T), indicating that the cell death here was associated with the mitochondrial apoptotic pathway. All the tested therapeutics delivered the promise of safety as reflected by the negligible death in hepatocytes. For comparison, we also analyzed the apoptosis profiles of liver tissues from host received treatment mediated by non-cleavable, Mito(T)-Nuc(T). Negligible cell death was witnessed in both cancer cells and hepatocytes (Supplementary Fig. 19). This finding implies that the non-cleavable platform only affects the cells minimally, independent of their types. As such, its therapeutic outcome, if any, is expected to be not much better than that of phosphate-buffered saline (PBS), making us exclude the non-cleavable platform from further assessment. The cell death induced by different therapeutics was further validated quantitatively via flow-cytometry. In this case, the expression of cleaved PARP, a biomarker indicative of end-stage apoptosis^43^, was evaluated. Those cancer cells were sorted according to their GFP expression, followed by the permeabilization and intracellular staining. As shown in Fig. 6b–d, significant cell death was noticed in the Mito(T)-pep-Nuc(T) group, which outperformed that of Mito(T)-pep-Nuc(N).

### Changes in gene expression

**Fig. 7:**
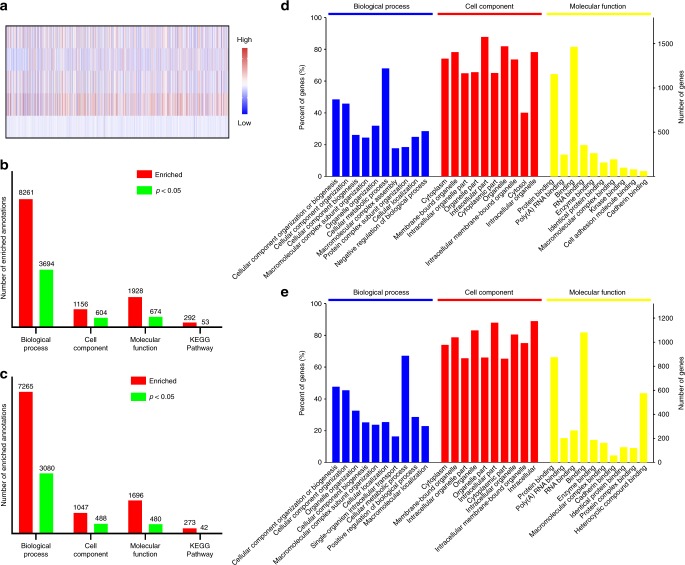
Changes in gene expression. **a** A heat map showing the expression levels of 5542 identified genes in liver metastasis lesions of mice received different treatments. Treatments (from top to bottom row): Mito(T)-pep-Nuc(T), Mito(T)-pep-Nuc(N), Mito(N)-pep-Nuc(T), Sorafenib, and PBS. **b**, **c** Charts showing the total number of enriched annotations for **b** up-regulated and **c** down-regulated genes whose differences in expression are of statistical significance. Four categories, including biological process, cellular components, molecular function, and KEGG pathway, were considered. **d**, **e** Top-10 significantly enriched GO annotations associated with **d** up-regulated and **e** down-regulated genes belonging to the term of Biological Process, Cell Component, and Molecular Function

We assessed the global changes in gene expression (Fig. 7). A map of total 5542 recognized genes was shown in Fig. 7a, and Gene Ontology (GO) analysis was conducted. Here, we focused only on engineered materials. It can be seen that more GO annotations were enriched for up-regulated genes (Fig. 7b) than down-regulated (Fig. 7c) ones, with top-10 annotations displayed in Fig. 7d, e, respectively.

Next, we analyzed the GO analysis results term-by-term. For biological process, both the up- and down-regulated genes in the Mito(T)-pep-Nuc(T) group were associated tightly with stress response (Supplementary Fig. 20a), whose significance was higher than that of the other two groups (Supplementary Figs. 21a and 22a). Meanwhile, the induction of apoptotic processes was uniquely found in the Mito(T)-pep-Nuc(T) group. As a matter of fact, the stress responses could take place long before apoptosis becomes necessary. Once the damage is beyond the cell’s capacity to repair, self-destruction could be iniated^44^. A closer association of several down-regulated genes with metabolic process manipulation in the Mito(T)-pep-Nuc(T) group (Supplementary Fig. 20b) than the other two (Supplementary Figs. 21b and 22b) validated the effectiveness of dual-targeting against a tumour. Also, the influence of Mito(T)-pep-Nuc(T) reached to several organelles (Supplementary Fig. 23a) to an extent more significant than it did by the other two (Supplementary Figs. 24a and 25a). We confirmed the association of down-regulated genes with cell-to-cell adhesion occurred during metastasis^45^ when both organelles were impaired (Supplementary Figs. 23b, 24b and 25b). To become metastatic, the subunits of microfilaments supporting the cancer cells undergo intense, ATP-dependent disassemble-assemble cycles^46^. Thereby, it is not hard to understand the depletion of ATP led to the loss of cell-to-cell adhesion (Supplementary Fig. 26). No similar implication was found in groups of Mito(T)-pep-Nuc(N) (Supplementary Fig. 27) and Mito(N)-pep-Nuc(T) (Supplementary Fig. 28), again revealing their limitations in globally influencing the cellular homeostasis. The KEGG enrichment revealed that Mito(T)-pep-Nuc(T) treatment interfered the protein engineering executed in the endoplasmic reticulum (ER), way stronger than that for DNA replication retardment caused by Nuc(T)-mediated intra-nuclear PTT (Supplementary Fig. 29a). Previous reports suggest that the execution of PDT or PTT might lead to the lethal accumulation of misfolded proteins in ER, referred to as unfolded protein response (UPR)^47^. Presumably, a particular population of those down-regulated genes (Supplementary Fig. 29b) might critically be linked to the elimination of misfolded proteins. A similar enrichment was witnessed in the up-regulated genes of the Mito(T)-pep-Nuc(N) group (Supplementary Fig. 30) but not in the Mito(N)-pep-Nuc(T) group (Supplementary Fig. 31), indicating that action of UPR relies more on the fueling of GLY than OXPHOS.

### Proteome study

**Fig. 8:**
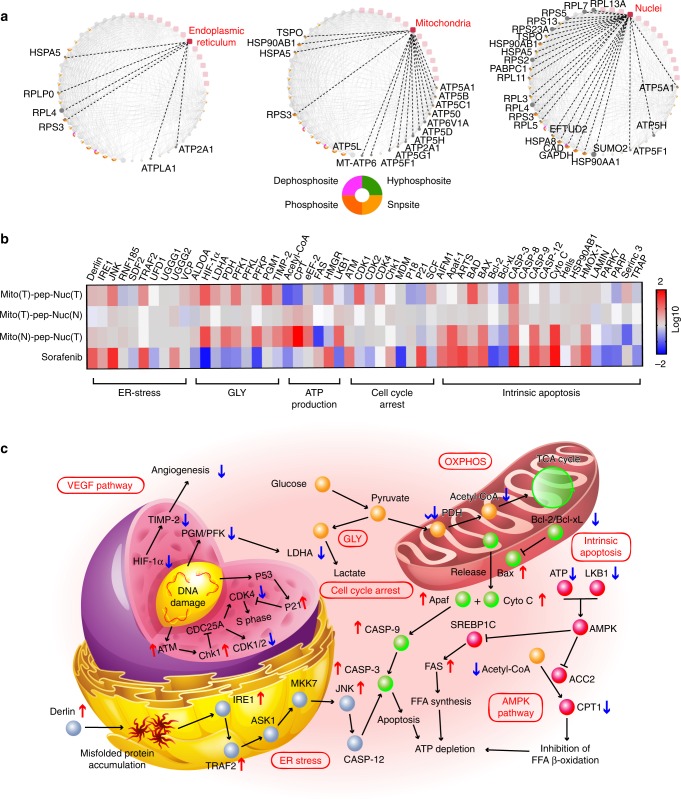
Proteome study. **a** Networks showing the corresponding down-regulated proteins identified by LC/MS that interact with three different organelles, including endoplasmic reticulum, mitochondria, and nuclei. Potential form of phosphorylation of each protein is shown below. **b** A heat map showing the enriched proteins that were associated with five classes of functions: ER stress, GLY, ATP production, cell cycle arrest, and intrinsic apoptosis. The bioinformatic analysis was conducted using Omicsbean Cancer software. A color bar on the right indicates the color scales. **c** A schematic illustrating the network of possible pathways underlying the therapeutic effect of Mito(T)-pep-Nuc(T) in cancer cells

Cellular compartments of these sorted cancer cells were labeled with tandem mass tag (TMT) before the analysis with mass spectra. Among the numerous recognized proteins, we focused on proteins clustered around mitochondrion, nucleus, and ER. Figure 8a shows the protein–organelle interaction networks. Down-regulation of ribosomal proteins, ER chaperone, and heat-shock protein (HSPA5) were confirmed. Furthermore, we found that mitochondria and nucleus were differently affected. In detail, the mitochondrial ATP production machinery was most vulnerable to treatment. In the nucleus, the results indicated that the activity of cell replication changed most considerably due to the depletion of pyrimidine, an essential substrate of nucleotide production^48^. Second and third to this were the functions of the ribosome and ubiquitination, respectively, whose abrogation promoted UPR.

Next, we analyzed these proteins that were functionally associated with biological processes including ER stress, GLY, ATP productions, and intrinsic apoptosis. Owing to the blocked DNA replication, an arrested cell cycle was expected, which was also evaluated here. A heat map was generated on this basis showing the changes of all related proteins (Fig. 8b), along with a scheme illustrating the anticipated pathways (Fig. 8c). It was found that both the ER stress and the release of cytochrome *C* can prime the cleavage of pro-Caspase 3 and eventually led to cell death. Notably, the AMPK (AMP-activated protein kinase) protein was inactivated in this case. Typically, the AMPK pathway could activate when facing the loss of ATP^49^. We found the up-regulation of liver kinase B1, an inhibitor of AMPK^49^, might rationalize the inactivation of AMPK. Besides, the silent AMPK allowed the production of fatty acid, through which the ATP was consumed further. Likewise, the up-regulated genes that positively associated with the lipid biogenesis also helped deplete the ATP storage.

### Attenuation of DLM **in vivo**

**Fig. 9:**
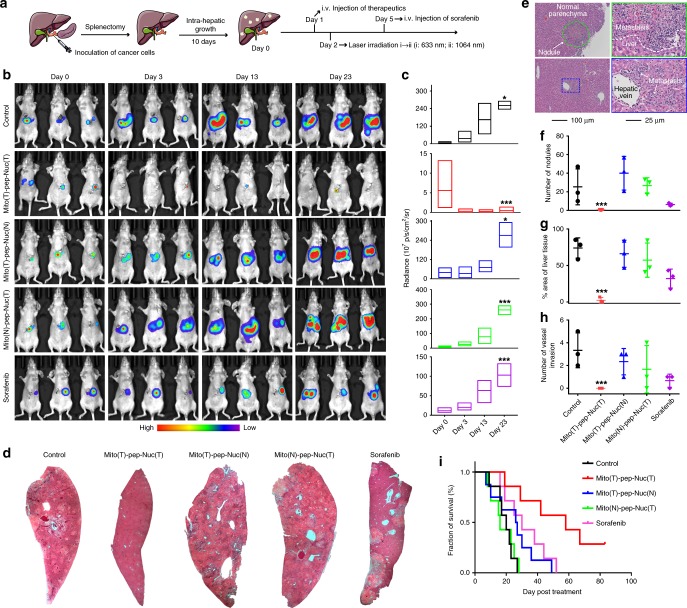
In vivo anti-metastasis effect. **a** A schematic showing the procedures of in vivo experiments (*n* = 7 in each group). **b** Bioluminescence graphs of mice at different time points after receiving the treatments with the radiance of liver tissues quantified and shown in **c** (*n* = 3). In the box plot, the upper and lower quartile as outlined by top and bottom boundary were divided by line showing the median value. **d** Necropsy of representative liver tissues collected at day 30 or the day they died if earlier than day 30. (A full spectrum of necropsy is shown in Supplementary Figs. 26–30). **e** Representative micrographs with H&E staining showing the pathological features of liver metastasis and pan-cancer normal parenchyma. The micrographs on the right panel show the details of regions that have been highlighted with either the green circle or blue box at a higher magnification. **f** Number of nodules that existed in an observation area (×10, average of ten fields for each mouse). **g** Area of the liver tissue that was occupied by metastasis. **h** The number of cases of invasion into liver vessels (*n* = 3). Median value was shown as line in the middle of the plot. **i** Survival profiles of mice received different treatments during an observation period of 80 days (*n* = 7). Data are presented as mean ± s.e.m. *P* values were calculated by one-way ANOVA (***p < 0.005, *p < 0.05)

Figure 9a shows the schedule of therapy; ten days were allowed for the onset of DLM, with a clear sign of metastasis nodule(s) formation in liver monitored by BLI (Fig. 9b, c). For the mice received PBS (control), the progress of metastasis was quick, which occupied the whole liver in 23 days. Expectedly, mice treated with Mito(T)-pep-Nuc(T) eliminated the metastasis lesions considerably (Fig. 9c). This also indicates that a vast majority of metastasis lesions are accessible to Mito(T)-pep-Nuc(T). Meanwhile, Mito(T)-pep-Nuc(N) or Mito(N)-pep-Nuc(T), though sort of effective at the very beginning, saw local relapse at rates even higher than that of control (Fig. 9b, c). We also noticed that sorafenib exerted a better therapeutic effect than either one of the half-targeting platforms (Fig. 9c), albeit long-term protection was not reached.

The mice were sacrificed for tissue collection and stained with hematoxylin and eosin (H&E). Figure 9d shows the representative micrographs of an individual lobe (for a full spectrum of them, please refer to Supplementary Figs. 32–36). Micronodules and local vessel invasion events were recorded (Fig. 9e) and quantified (Fig. 9f–h). In the Mito(T)-pep-Nuc(T) group, metastasis lesion decreased in number and volume, thus favoring an improved prognosis (median survival: 55 days versus 19 and 29 days for control and sorafenib groups, respectively, Fig. 9i). By contrast, Mito(N)-pep-Nuc(T) promoted the generation of more metastasis nodules (Fig. 9f), wherein a poor prognosis was witnessed (Fig. 9i).

### Assessments of compatibility

**Fig. 10:**
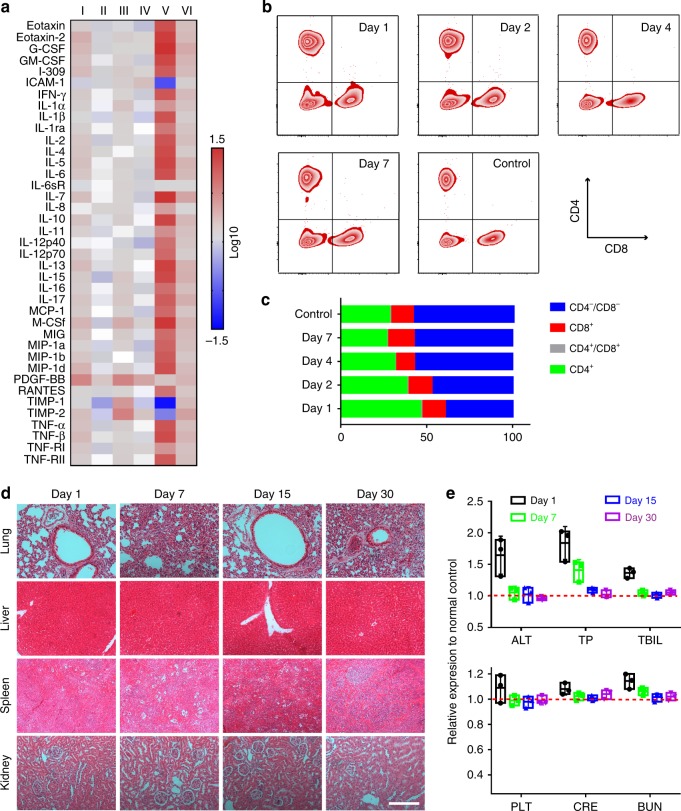
Long-term safety profile. **a** A heat-map showing the changes in the expression of a spectrum of inflammatory cytokines in the liver tissue (*n* = 3 at each time point). Their levels of expression were first analyzed using a protein antibody array, normalized to that of the normal control, and shown in a fold-of-change fashion. Captions I–VI indicate the case of mice received Mito(T)-pep-Nuc(T) (I, II), Mito(T)-pep-Nuc(N) (III, IV), Mito(N)-pep-Nuc(T) (V, VI) at Day 1 (I, III, V) and Day 15 (II, IV, VI) as defined in Fig. 8a. A color bar on the right indicates the color scales. **b** Profile of systematic pro-inflammatory immunity in responding to Mito(T)-pep-Nuc(T)-mediated therapy. The peripheral level of CD4^+^ and CD8^+^ T cells were analyzed by flow cytometry, and **c** the population of all four different classes of circulating T cells including CD4 and CD8 dual-positive (CD4^+^/CD8^+^) and dual-negative (CD4^−^/CD8^−^) and singly positive ones (CD4^+^ and CD8^+^). **d** Pathological study of normal mice received i.v. injection of Mito(T)-pep-Nuc(T). The H&E-stained tissue slices of four organs were shown as a function of time post injection. Scale bar: 100 μm. **e** Hematological study showing the functions of liver (upper), spleen, and kidney (lower) as a function of time as indicated by the biomarkers for liver (including ALT, TP, TBIL), spleen (PLT, platelet), and kidneys (including BUN (blood urea nitrogen) and CRE (creatinine)). Data are presented as mean ± s.e.m. (*n* = 3). In the box plot, the upper and lower quartile as outlined by top and bottom boundary was divided by line showing the median value

The safety concern of our platform was addressed in different aspects. First, we assessed the inflammatory reaction. The mice carrying DLM were treated prior to sacrifice on Days 3 and 15 as described in the schedule (Fig. 9a), followed by the extraction of the hepatic fluid. Then, the expression level of a variety of cytokines was measured as shown in the heat map (Fig. 10a). Only weak inflammation, as marked by the increased expression of G-CSF, I-309, IL-13, and PDGF-BB, took place in the Mito(T)-pep-Nuc(T) group (Day 3, I). The inflammation was temporal and weakened within the following days (Day 15, II). Likewise, treatment mediated by Mito(T)-pep-Nuc(N) (Fig. 10a), but not Mito(N)-pep-Nuc(T), also resulted in hepatic inflammation, which recovered transiently. Up-regulation of an overwhelming portion of the tested cytokines revealed that the Mito(N)-pep-Nuc(T) remodeled the niche toward one favoring the cancer progress. It is thereby easy to understand the suboptimal prognosis in the case of Mito(N)-pep-Nuc(T).

Next, we assessed the changes in systematic immunity caused by treatment mediated by Mito(T)-pep-Nuc(T). To this end, flow-cytometry was leveraged to measure the populations of CD3^+^ T cells^50^. Specifically, the population of CD4^+^ T cells increased from 28.3% (control) to 46.3% at Day 1 (Fig. 10b, c) and gradually declined to 26.6% on Day 7. The mitigated inflammation was also reflected by the changes in CD8^+^ T cell populations (Fig. 10c). Altogether, the evidence is enough to support the assumption that the host is well tolerable with the temporal inflammatory response induced by therapy.

Furthermore, we confirmed that prolonged exposure to Mito(T)-pep-Nuc(T) led to negligible complications (Fig. 10d). The promise of safety was also assessed by hematology results (Fig. 10e). Functions of the liver were minorly affected during the early exposure (Day 1). Stress was induced and relieved gradually thereafter. Besides, we found that either the spleen or kidney was vulnerable to the treatment, which can be explained by the limited accumulation of nanoparticles in these organs as verified by the pharmacokinetic study.

## Discussion

The concept of targeted delivery at precision down to organelle level has emerged for a while^51–53^, the action, however, is detrimental to both cancerous and healthy cells in most cases. As such, delivery strategies harnessing targeting moieties are favorably used in the hope of making lesions the only site for the drug to accumulate. To effectively treat DLM while sparing abundant normal cells, alternative methods beyond active targeting delivery should be employed. Attempts have already been made with a similar aim to confine the action in cancer cells by harnessing the ubiquitous pathologic features tumour develops^54,55^. So far, success in DLM model remains elusive, in which the platform should be reconfigured by taking a full consideration of the sophisticated liver niche. We demonstrated that the concurrent interruption of GLY and OXPHOS activities in cancer cells could effectively eliminate liver metastasis while sparing hepatocytes. WONPs play dual roles as both SOS-scavenger and PTT agent. Once optimized, the SOS produced during PDT could be stoichiometrically depleted by WONPs, and in turn, be oxidized entirely into PTT-inert species. Such a feature meets our expectation of reaching a termination between PDT and PTT. Another crucial parameter is the choice of the enzyme for cleaving the Mito(T)-pep-Nuc(T). Given the aberrantly high expression of Cathepsin B in cancer cells, the peptide cleavable by this enzyme was selected to link Nuc(T) and Mito(T).

In summary, a metabolism-based, tumour-selective therapeutic strategy was demonstrated here to conquer liver metastasis by harnessing an energy depletion mechanism. Their interaction with cellular components and influences on the metabolic manners of cells have been studied. Further proof-of-concept results, including the profiles of survival and inflammatory response, confirmed the effectiveness and compatibility of our strategy. Given the abnormal expression of Cathepsin B enzyme in a spectrum of tumours, as well as the ubiquitous metabolic phenotype of cancer cells, the strategy demonstrated here could have implications in cancers of various types.

## Methods

### Materials

Tungsten hexachloride (WCl_6_, ≥99.9%), (4-carboxybutyl)triphenylphosphonium bromide (TPP, 98.0%) (3-aminopropyl)triethoxysilane (APTES, ≥99.0%), poly(acrylic acid) (PAA, M_W_~2 kDa), diethylene glycol (DEG, ≥99.0%), ethylenediamine (EDA), cysteamine (≥95.0%), *N*-(3-dimethylaminopropyl)-*N*′-ethylcarbodiimide hydrochloride (EDC, Bioxtra), *N*-hydroxy-succinamide (NHS, 98.0%), and chlorpromazine (≥98.0%) were all obtained from Sigma-Aldrich (United States). Tetraethyl orthosilicate (TEOS), hexadecyl trimethyl ammonium bromide (CTAB), hydrogen peroxide (H_2_O_2_), and ethanol were all purchased from J&K Scientific (P. R. China) that were all of the analytic grades and used without further purification. Chlorin e6 (Ce6,≥99.0%) was purchased from Cayman Chemical (United States). The Cathepsin B-cleavable peptide (NHS-Gly-Phe-Lys-Phe-Lys-Gly-maleimide, ≥97.0%) and nuclear localizing sequence (NLS, CGGGPKKKRKVGG-maleimide, ≥99.0%) were synthesized by Yarebio (P. R. China). Recombinant proteins of Caspase 3, MMP-2, and Cathepsin B protein were all purchased from R&D Systems (United States). The inhibitors of Caspase 3, MMP-2, and Cathepsin B, Ac-DEVD-CHO, SB-3CT, and CA-074 methyl ester, respectively, and sorafenib (≥99.9%) were obtained from Selleckchem (United States). Modified McCoy’s 5a medium was obtained from American Type Culture Collection (ATCC, United States). Dulbecco’s modified Eagle’s medium (DMEM), fetal bovine serum (FBS), PBS, penicillin/streptomycin, and H_2_DCFDA probe were obtained from Invitrogen (Thermo Fisher Scientific, United States). d-Luciferin potassium salt (≥95.0%) was purchased from Sciencelight (P. R. China). Deionized (DI) water produced with a Millipore ultrapure water system (United States) featuring a resistivity of 18.2 MΩ cm and was used throughout the experiments.

### Cell lines

The HCT-116, a human primary colon cancer cell line, was purchased from ATCC (CCL-247). Human immortalized hepatocytes, HHL-5, were obtained from Culture Collection of the Chinese Academy of Sciences (Shanghai, P. R. China). HCT-116 cells were cultured and maintained in modified McCoy’s 5a medium supplemented with 10% FBS and 1% penicillin/streptomycin. HHL-5 cells were cultured under an identical condition except for the culture medium that was replaced by DMEM. Cell cultures were maintained in an incubator at 37 °C in a humidified atmosphere with 5% CO_2_. The medium was replaced every other day until the confluency of cells was in the range of 80–90%. For the in vivo and part of the in vitro analyses, the HCT-116^*GFP/Luc*^ cells were used. They were established through transfection with lentivirus vectors carrying the plasmids of both firefly luciferase and GFP. The obtained HCT-116^*GFP/Luc*^ cells were cultured in modified McCoy’s 5a medium supplemented with 20% FBS and 1% penicillin/streptomycin. Likewise, human HCCcells, HCC-LM3 (obtained from Shanghai Zhongshan Hospital), were transfected to express GFP, giving the HCC-LM3^*GFP*^ cells. They were cultured in DMEM supplemented with 20% FBS, 1% penicillin/streptomycin, and 1% (vol/vol) mycoplasma inhibitor (Biological Industries, Israel). The mouse monocyte, RAW 264.7 cells (obtained from ATCC), were cultured in DMEM supplemented with 10% FBS and 1% penicillin/streptomycin.

### Mouse model of liver cancer

Female BALB/c *nu*/*nu* nude mice (4–6 weeks old) were purchased from the Comparative Medicine Centre of Yangzhou University and raised in a specific pathogen-free (SPF) facility. The liver metastasis was established based on a published protocol with minor modifications. In brief, HCT-116 cells were allowed to grow in a 75-cm^3^ plate until a 90% confluency was achieved before their digestion and resuspension in the serum-free culture medium. Afterward, the spleen of the mouse was exposed under sterilized condition through the incision on its left flank, followed by the titration of 1 × 10^6^ cells suspended in 50 μL of medium. The splenectomy was conducted at 2 min later. In order to establish the HCC animal model, the left lobe of the nude mouse was exposed surgically, with a total of 2 × 10^6^ HCC-LM3^*GFP*^ cells administrated within 30 s. Afterward, a cotton bud was pressed gently on the injection site before the needle was retracted, and kept still for another 2 min in order to stop the bleeding.

In order to eradicate the liver metastasis, different therapeutics were leveraged. For the synthesized hybrid nanomedicine, including Mito(T)-pep-Nuc(T), Mito(T)-pep-Nuc(N), and Mito(N)-pep-Nuc(T), their compositions were optimized as described in the text, and suspended in PBS prior to their systematic administration into mice carrying liver metastasis (dosage: 15 mg per mouse) at Day 10 post the inoculation of HCT-116^*GFP/Luc*^ cells (15 mg per mouse, *n* = 7 in each group). After 24 h, an incision (5 cm in length) in parallel to the longitudinal direction of the liver was made to expose the whole tissue under the sterilized condition, followed by the laser irradiation. In a typical cycle of treatment, the power densities of PDT and PTT were kept at 1, and 0.75 W/cm^2^ for a duration of 15 and 10 min, respectively, and the irradiation of the whole liver tissue can be finished with three cycles of subsequent irradiation. During this process, the mice were all kept anesthetized using inhalation anesthesia by isoflurane. For sorafenib, no laser irradiation was executed after the systematic injection of therapeutics suspended in PBS (dose regimen: 25 mg kg^−1^ weight, *n* = 7). This was repeated every 4 days (as illustrated in Fig. 8a on Day 1 and Day 5).

All the protocols for the animal tests have been reviewed and approved by the Committee on Animals at Nanjing University and performed in accordance with the guidelines provided by the National Institute of Animal Care.

### Synthesis

Briefly, the W_18_O_49_ nanoparticles encased by PAA were prepared using a polyol method by taking WCl_6_ and DEG as the metal precursor and the reducing agent in addition to its role as the solvent, respectively^34^. Their thiolation was realized through the conjugation of cysteamine with PAA-functionalized W_18_O_49_ nanoparticles via EDC/NHS. The as-prepared, thiol-terminated W_18_O_49_ nanoparticles were covalently conjugated with NLS peptide. In a typical experiment, 15 mg of the synthesized NLS peptide was dissolved in 2 mL PBS containing thiol-functionalized W_18_O_49_ nanoparticles (1 × 10^18^ particles per mL), and the covalent binding between the thiol group and maleimide was allowed to proceed at 4 °C overnight that was protected from direct light exposure. Afterward, the NLS-functionalized W_18_O_49_ nanoparticles, denoted by Nuc(T), was suspended in PBS to a final concentration of 2 × 10^17^ particles per mL. For the Mito(T), it was prepared at room temperature by harnessing the EDC/NHS chemistry. Typically, MSNs were firstly modified with APTES by following a published method^33^, and TPP (13.2 mg) was dissolved in 5 mL ethanol suspension of APTES-functionalized MSNs (1 × 10^17^ particles), along with 10 μL of EDA to facilitate the coupling between the carboxyl group of TPP and amine of APTES. After a 4 h reaction under an ambient condition, the TPP-functionalized MSNs were harvested via centrifugation and were purified through repeated washing with DI water. Next, different amounts of photosensitizer, Ce6, were loaded into the interior of MSNs through covalent binding with APTES as reported^56^. The Ce6-loaded Mito(T) were collected and washed as mentioned above, before re-suspending in PBS to a final concentration of 5 × 10^15^ particles per mL. The Cathepsin B-cleavable peptide (30 mg) was conjugated to the synthesized Mito(T) through the coupling between NHS and the excess amine groups of Mito(T), followed by their conjugation with Nuc(T) in a fashion similar to what was used for the functionalization of W_18_O_49_ nanoparticles with NLS peptide.

Fluorescent labeling of Nuc(T) was conducted, if necessary, prior to their coupling with enzyme-cleavable peptide by following our published protocol with slight modification^34^. Specifically, Alexa Fluor-488-maleimide (Thermo Fisher Scientific, 0.5 mg mL^−1^ in PBS) was used to label Nuc(T) (5 × 10^17^ particles per mL) according to the manufacturer’s instructions. The dye-labeled Nuc(T) nanoparticles were purified via centrifugation and repeatedly washed with PBS to remove non-specifically absorbed dyes.

### Characterization

The morphology of nanoparticles was investigated by TEM (TEM-200CX, JEOL, Japan) and HRTEM (TEM-2100, JEOL) The UV–vis–NIR spectra of nanoparticles were recorded on a UV-vis spectrophotometer (UV3100, Shimadzu, Japan). The Zeta-potential, as well as the DLS profiles of corresponding nanoparticles, was measured at room temperature using a Zeta-sizer (Nano ZS, Malvern, United Kingdom). Surface element composition was investigated by XPS (K-Alfa, Thermo Fisher Scientific, United States) with a focused monochromatic Al X-ray (1486.6 eV) source. The concentration of tungsten in each sample was determined by ICP-MS (Agilent 7500ce, Agilent, United States).

### Flow-cytometry analysis

For the evaluation of the intracellular expression level of ROS, both HCT-116 and HHL-5 cells were seeded in a six-well plate and cultured until a confluence of 80% was reached. Next, the culture medium was replaced by a fresh one containing Mito(T)-pep-Nuc(T) (Ce6: 2.13 mM; Nuc(T): 2.35 × 10^15^ particles per mL). The incubation was allowed to proceed for 4 h under the condition generally used for cell culture, followed by three washes with warm PBS to remove excess nanoparticles. Next, the cells received sequential laser irradiations ((i**)** 633 nm at a power density of 1 W cm^−2^ for 15 min; (ii) 1064 nm at a power density of 0.75 W cm^−2^ for 10 min). The intracellular level of ROS was evaluated using a ROS-responsive probe, H_2_DCFDA (500 μL, 1 mM). After 10 min, the ROS-associated fluorescence intensity was analyzed using flow-cytometry (FACS verse; BD Bioscience, United States), and the data were processed using FlowJo software (version 10.0.7).

For the quantification of apoptosis profiles of cancer cells metastasized to liver, mice in different groups were sacrificed at 6 h post the second-round laser irradiation (for sorafenib, the time point was extended to 12 h post its systematic administration), with their liver tissues collected and minced into pieces with a mean volume of 5 mm^3^ using an eye scissors. These tissue fragments were immediately transferred to DMEM culture medium supplemented with 30 μg of type IV collagenase (Sigma-Aldrich) and type I DNase (Roche) and incubated for 2 h at 37 °C. After the tissue fragments were softened, they were subjected to mincing with a 400-mesh screen to acquire a suspension of single cells differ in type. After the centrifugation (200*g* for 5 min), the cell debris in the supernatant was discarded, leaving only the intact cells collected and resuspended in PBS. For the cancer cells in the mixture, they were sorted by flow-cytometry on the basis of their expression of GFP. The as-obtained metastatic cancer cells typically have purities in the range of 88–95% and were used directly for down-stream assays if necessary. Otherwise, they were cryopreserved using a freezing container at −80 °C (Nalgene, United States).

For the quantification of nanoparticles accumulated in cancer and Kupffer cells, mice bearing either HCC or DLM were injected intravenously with Mito(T)-pep-Nuc(T) (dosage: 15 mg per mouse), followed by sacrifice at different time points to harvest a serial of organs including liver, spleen, lungs, kidneys, in addition to blood sample. The contents of tungsten were measured using ICP-MS. To assess the accumulation difference between cancer and Kupffer cells, the liver tissues collected at 8 h post the injection were processed into single-cell suspension, with cancer and Kupffer cells sorted according to their expression of GFP and F4/80 (labeled 1h prior to the sorting using antibodies, clone: BM8, dilution: 1:100; Invitrogen). Changes in the Ce6 expression level in those sorted cells were analyzed and calculated by normalizing the measured values to that of HCC-LM3 and RAW 264.7 cells received no treatment for cancer and Kupffer groups, respectively.

For the analysis of population of different classes of CD3^+^ T cells in peripheral blood of mice received the treatment mediated by Mito(T)-pep-Nuc(T), 100 μL of whole blood were extracted and treated with ACK Lysis Buffer (BD Biosciences) followed by surface staining with FITC-labeled rat-anti-mouse CD8a (clone: 53-6.7, dilution: 1:50) and PE-labeled rat-anti-mouse CD4 antibodies (clone: RM4-5, dilution: 1:20). The FITC-labeled rat IgG2a and PE-labeled rat IgG2a were selected as isotypes (dilution: 1:50). All these antibodies were purchased from BD Biosciences and used according to the manufacturer’s instructions.

### Measurements of OCR and ECAR

The cells, both normal and cancerous, were seeded in a special 24-well plate suitable for the Seahorse XF-24 Extracellular Flux Analyzer (Agilent Technologies) at a density roughly the same as the one achieved by using a traditional 24-well culture plate. Afterward, the cells were incubated with different nanoparticles and sequentially treated as described above for Mito(T)-pep-Nuc(T). The status of both OXPHOS and GLY have been evaluated at 1 h post the second-round laser irradiation, with the chemical efforts sequentially introduced as detailed in both Fig. 4a and a previous publication^40^.

### Fluorescence imaging

In order to assess the subcellular distribution of Mito(T)-pep-Nuc(T) after their uptake by cells (the protocol was the same as the one used for the evaluation of the ROS level in cells using flow-cytometry), the structure of mitochondria or nucleus was stained with MitoTracker Green or Hoechst 33342 (Thermo Fisher Scientific), respectively, for 10 and 5 min under an ambient condition free from direct light irradiation. The fluorescence micrographs were collected on a fluorescence microscope (BX51, Olympus, Japan) and processed using a Bitplane Imaris software (version 7.4.2). For the time-lapse study, the fluorescence microscope was equipped with a live cell imaging chamber that was coupled with a gas controller (ibidi, Germany). In this case, the cells were placed in the environment containing 5% CO_2_ with the temperature set at 37 °C throughout the imaging. To understand how the enzymatic activities of Caspase 3, MMP-2, and Cathepsin B, as well as the presence of Clathrin influence the subcellular distribution of Nuc(T), they were blocked at 2 h prior to the incubation with Mito(T)-pep-Nuc(T) with Ac-DEVD-CHO (final concentration: 0.2 nM), SB-3CT (final concentration: 14 nM), CA-074 methyl ester (final concentration: 8 μM), and chlorpromazine (10 μg mL^−1^), respectively. All these inhibitors were solubilized in DMSO before their introduction into the culture medium of the cell.

### Bioluminescence imaging

The onset/development of liver metastasis was monitored using BLI. At each time point, the mouse received an intraperitoneal injection of a PBS solution containing d-luciferin (200 mg kg^−1^). After 20 min, the mouse was anesthetized with isoflurane via inhalation and imaged using an IVIS 200 system (PerkinElmer Imaging Systems, United States). Data were processed and reconstructed on the workstation.

### Immunofluorescent imaging

Freshly collected liver tissue was first embedded in an optimal cutting temperature compound (Tissue-Tek, Sakura, United States) and frozen on ice. Frozen blocks were sectioned to a thickness of 10 μm and mounted onto glass slides. Blocking and hybridization were performed in 3.0% (wt/vol) BSA in PBS. All images were acquired and processed the same as we did for fluorescence imaging.

### Long-term cytotoxicity

Antibody array assays were leveraged to evaluate the inflammatory response (Inflammation cytokine array; Ray Biotech Inc., United States) and were conducted according to the manufacturer’s instructions. In this case, the liver metastasis bearing mice were sacrificed at Days 1 and 15 after the treatments and minced into single-cell suspension using a 400-mesh screen within 1 h after the removal of the liver tissue. The suspension containing both body fluid and cells was centrifugated, and the supernatant containing the inflammatory cytokines was collected and stored at −80 °C prior to the microarray assay. For the working principles as well as the data collection and subsequent analysis, please refer to our previous publication^34^.

Normal female nude mice that had received an i.v. injection of Mito(T)-pep-Nuc(T) nanoparticles (15 mg per mouse) were sacrificed on Days 1, 7, 15, and 30 post the injection. Both the pathological and hematological analyses were conducted to assess the potential systematic toxicity caused by Mito(T)-pep-Nuc(T). Specially, for pathological studies, four major organs, including lungs, liver, spleen, and kidneys, were harvested at each time point, with their sections stained with H&E. For the hematological study, peripheral blood was collected and stored in a sodium EDTA anticoagulant tube (BD Bioscience, United States). Potential cytotoxic and allergic effects were reflected by the variation of ALT, TP, total bilirubin TBIL level, platelet, BUN, and creatinine (CRE) expressions.

To assess the potential complications associated with PDT alone, the mice received the systematic injection of Mito(T)-pep-Nuc(T) (15 mg per mouse) were irradiated with 633-nm laser (1 W cm^−2^, 15 min).

### Analyses of changes in gene and protein expression

Cancer cells metastasized to the liver were collected via the flow-cytometry sorting, followed by their extraction of total RNA and proteins that serve as the materials for gene expression and proteome analyses, respectively. The changes in gene expression were evaluated using a microarray (SurePrint G3 array v2.0 8 × 60k for human; Agilent Technologies, United States) according to the manufacturer’s instructions. The changes of protein expression were assessed using quantitative LC/MS based on TMT-labeling (a coupled system of QExactive mass spectrometer and the Easy Nano LC; Thermo Fisher Scientific). Peptides were detected with a well-established top-20 method in an Orbitrap mass spectrometer equipped with a hybrid dual-cell quadrupole linear ion trap (LTQ Orbitrap Elite; Thermo Fisher Scientific). The obtained MS2 spectra were searched using the SEQUEST algorithm (version 28)^57^, with the possible positively expressed proteins listed, whose sequences matched with that of those existed human-originated proteins (*Homo sapiens*) as displayed in the database. By taking a collective consideration of false discovery rate and *p* value calculated based on independent tests, those proteins with changes in expression levels as reflected by absolute fold change greater than 1.2 were chosen for bioinformatic analyses. For the differently expressed genes or proteins, GO analysis was performed using OmicsBean Cancer in order to better understand the molecular mechanism underlying the therapeutic effect exerted by different treatments.

### Statistics

Triplicate data were analyzed with Student’s *t-*test and one-way ANOVA using GraphPad Prism software (version 7.0); the significance level was set at *p* < 0.05. Significant statistical differences are indicated by asterisks in corresponding figures.

### Reporting Summary

Further information on research design is available in the Nature Research Reporting Summary linked to this article.

## Supplementary information

Supplementary info

Reporting Summary

## Data Availability

The authors declare that the data supporting the findings of this study are available in the article and associated Supplementary Information. Extra data or information are available from the corresponding authors upon reasonable request.
