# Supplementary material for: Eradication of unresectable liver metastasis through induction of tumour specific energy depletion
Source: Nat Commun. 2019 Jul 11;10:3051. doi: 10.1038/s41467-019-11082-3 (PMC6624273; doi:10.1038/s41467-019-11082-3)
Supplement: Supplementary file 1 — Supplementary info [file 41467_2019_11082_MOESM1_ESM.docx]

Supporting information

**Eradication of unresectable liver metastasis through induction of tumour specific energy depletion**

Da Huo^1^, Jianfeng Zhu^1^, Guojun Chen^2,3^, Qian Chen^2,3^, Chao Zhang^1^, Xingyu Luo^1^, Wei Jiang^1^, Xiqun Jiang^4^, Zhen Gu^2,3^ & Yong Hu^1^

**^1^** Collaborative Innovation Center of Chemistry for Life Sciences, College of Engineering and Applied Sciences, Nanjing University, Nanjing, Jiangsu, China. **^2^** Department of Bioengineering and the California Nanosystems Institute, University of California, Los Angeles, CA, United States. **^3^** Jonsson Comprehensive Cancer Center and Center for Minimally Invasive Therapeutics, University of California, Los Angeles, CA, United States. **^4^** Department of Polymer Science & Engineering, College of Chemistry & Chemical Engineering, Nanjing University, Nanjing, Jiangsu, China. Correspondence and requests for materials should be addressed to X.Q.J. (email: [jiangx@nju.edu.cn](mailto:jiangx@nju.edu.cn)), Z.G. (email: guzhen@ucla.edu), or Y.H. (email: hvyong@nju.edu.cn)


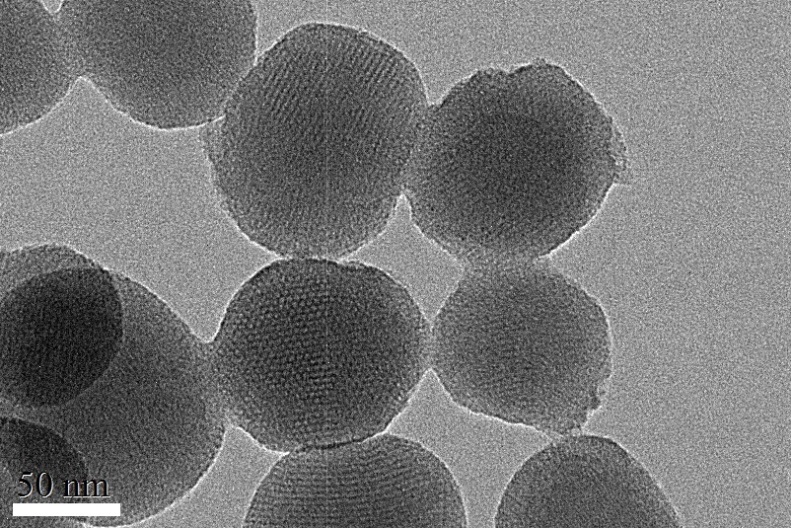


**Supplementary Figure 1.** TEM micrograph of synthesized MSNs. Scale bar: 50 nm.


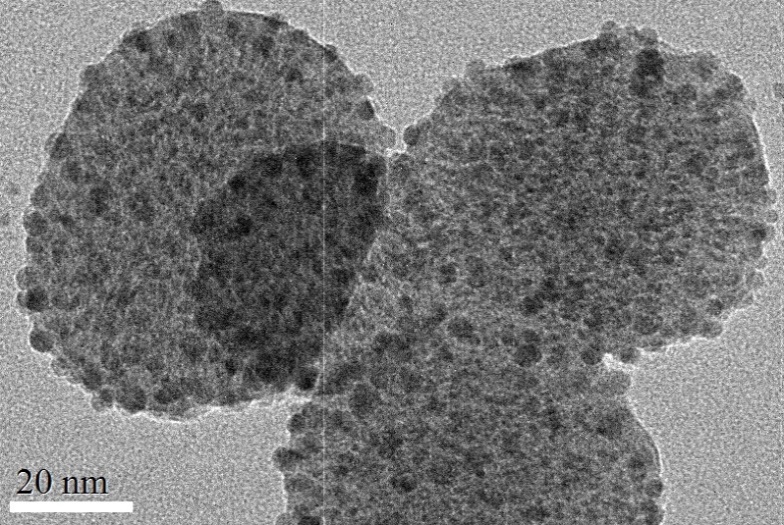


**Supplementary Figure 2.** HRTEM micrograph of representative Mito(T)-pep-Nuc(T) nanoparticles. Scale bar: 20 nm.


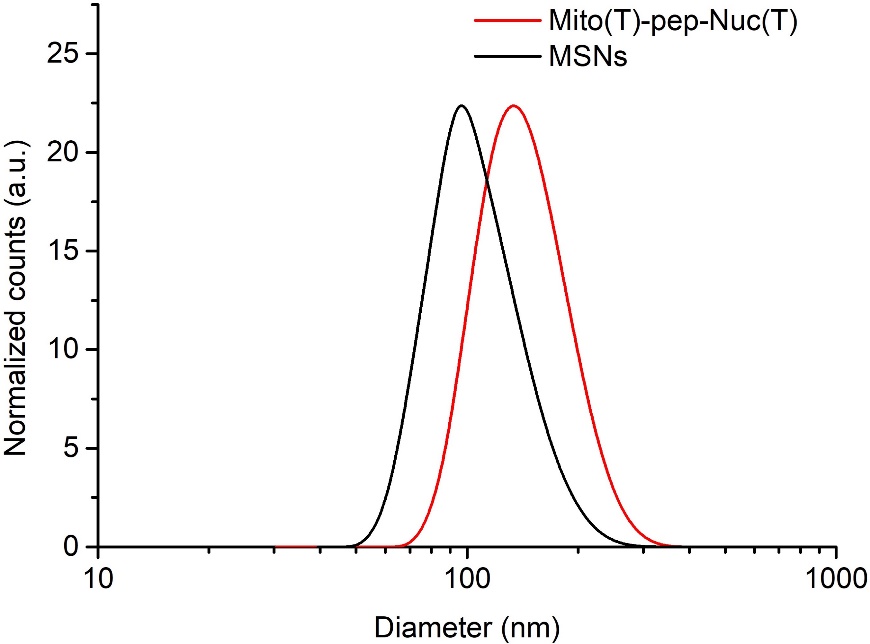


**Supplementary Figure 3.** The DLS results showing the size variation induced by the conjugation of WONPs onto MSNs.


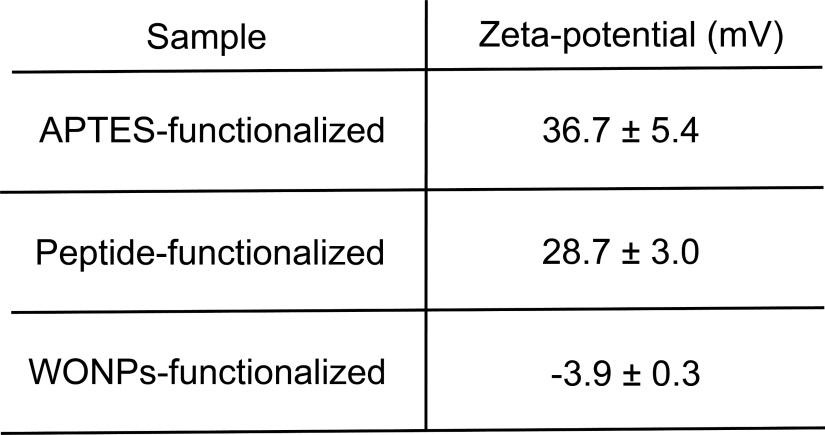


**Supplementary Figure 4.** Zeta-potential profiles of three nanoparticles emerged during the preparation of Mito(T)-pep-Nuc(T), sequentially denoted by APTES-, peptide-, and WONPs-functionalized.


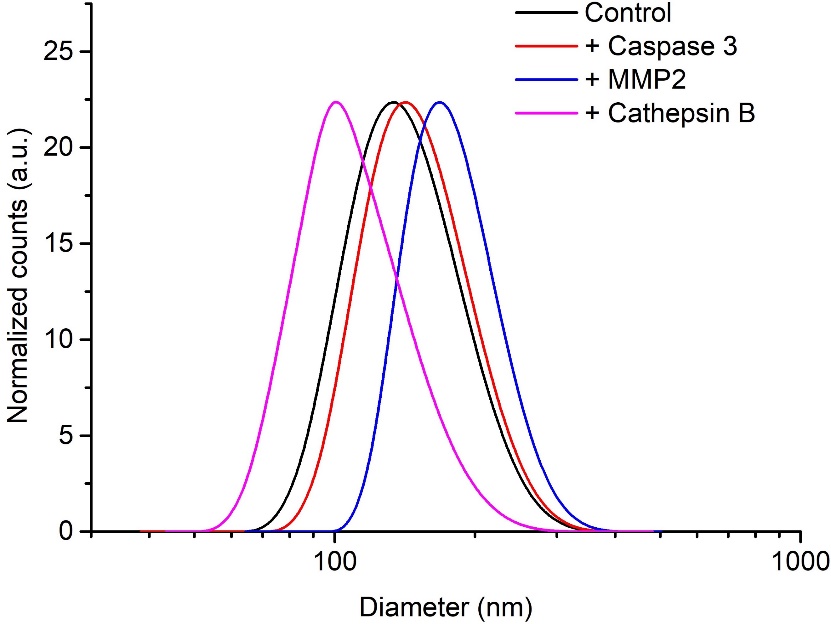


**Supplementary Figure 5.** The DLS results showing the size variation of Mito(T)-pep-Nuc(T) caused by the enzymatic effect of three classes of enzymes, including Caspase 3, MMP-2, and Cathepsin B.


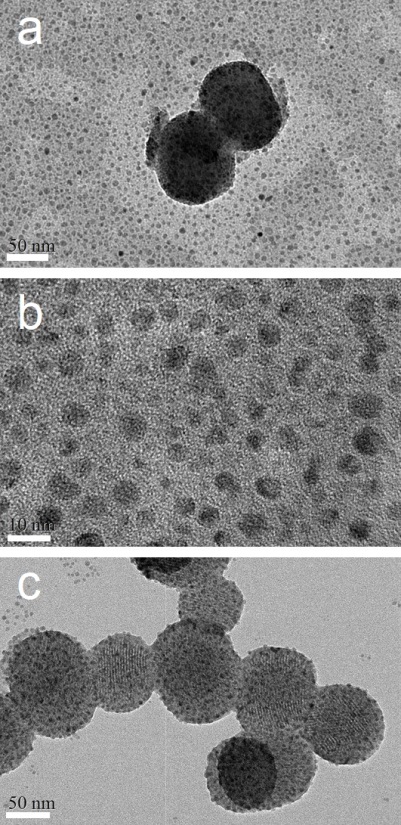


**Supplementary Figure 6.** (a) TEM micrograph of Mito(T)-pep-Nuc(T) post incubation with Cathepsin B. In this case, 2 μL of the suspension containing both the MSNs and released WONPs was dropped directed on the copper grid that was covered by a thin layer of carbon film. Scale bar: 50 nm. (b) HRTEM micrograph of released WONPs. Scale bar: 10 nm. (c) TEM micrograph of Mito(T)-pep-Nuc(T) post incubation with Caspase 3. Scale bar: 50 nm.


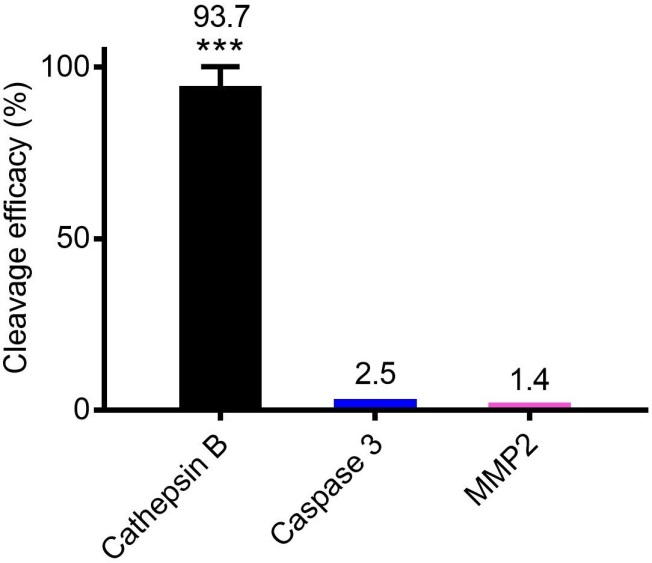


**Supplementary Figure 7.** The cleavage efficacy of peptide linking MSNs and WONPs by different enzymes. It was calculated on the basis of WONPs that were detached from MSNs after the incubation with certain enzymes. The content of tungsten remaining on the Mito(T)-pep-Nuc(T) was measured using ICP-MS. Data are presented as mean ± s.e.m. (n=3). *P* values were calculated by One-Way ANOVA (****p*<0.001).


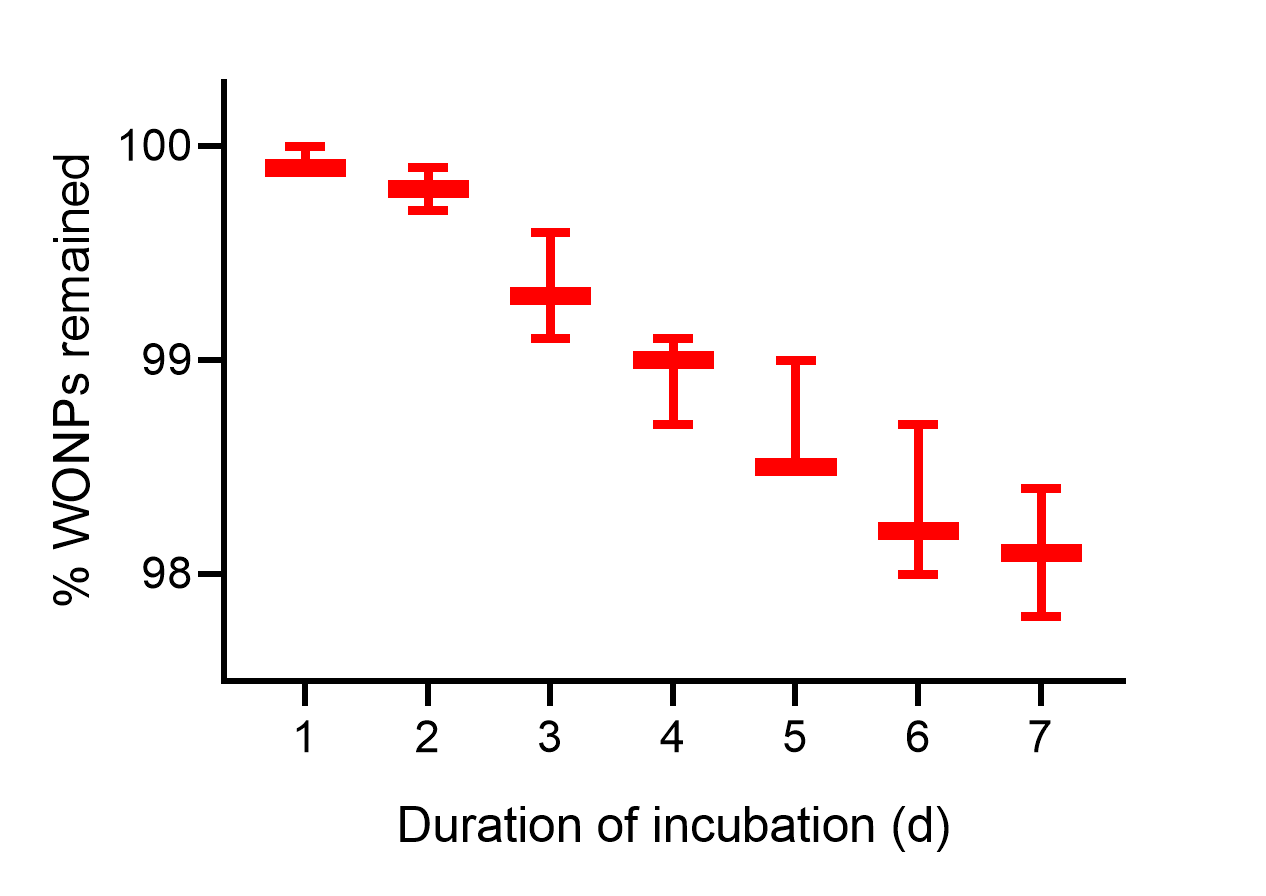


**Supplementary Figure 8**. Cumulative detachment of WONPs from the Mito(T)-pep-Nuc(T) platform in a course of 7 days. Data are presented as mean ± s.e.m. (n=3).


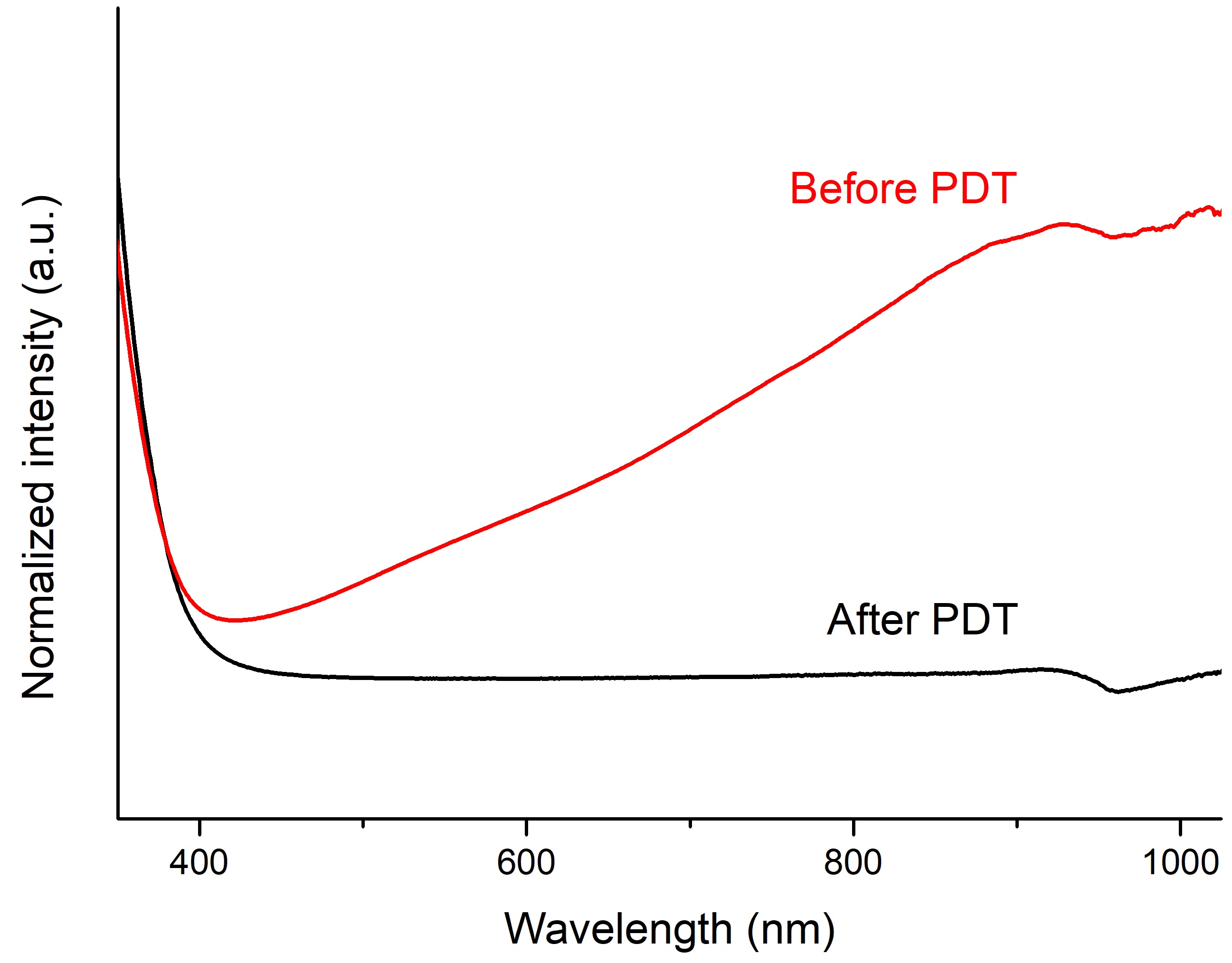


**Supplementary Figure 9**. The UV-vis-NIR spectra of MSN-pep-WONPs before and after 633-nm laser irradiation (1 W/cm^2^, 15 min).


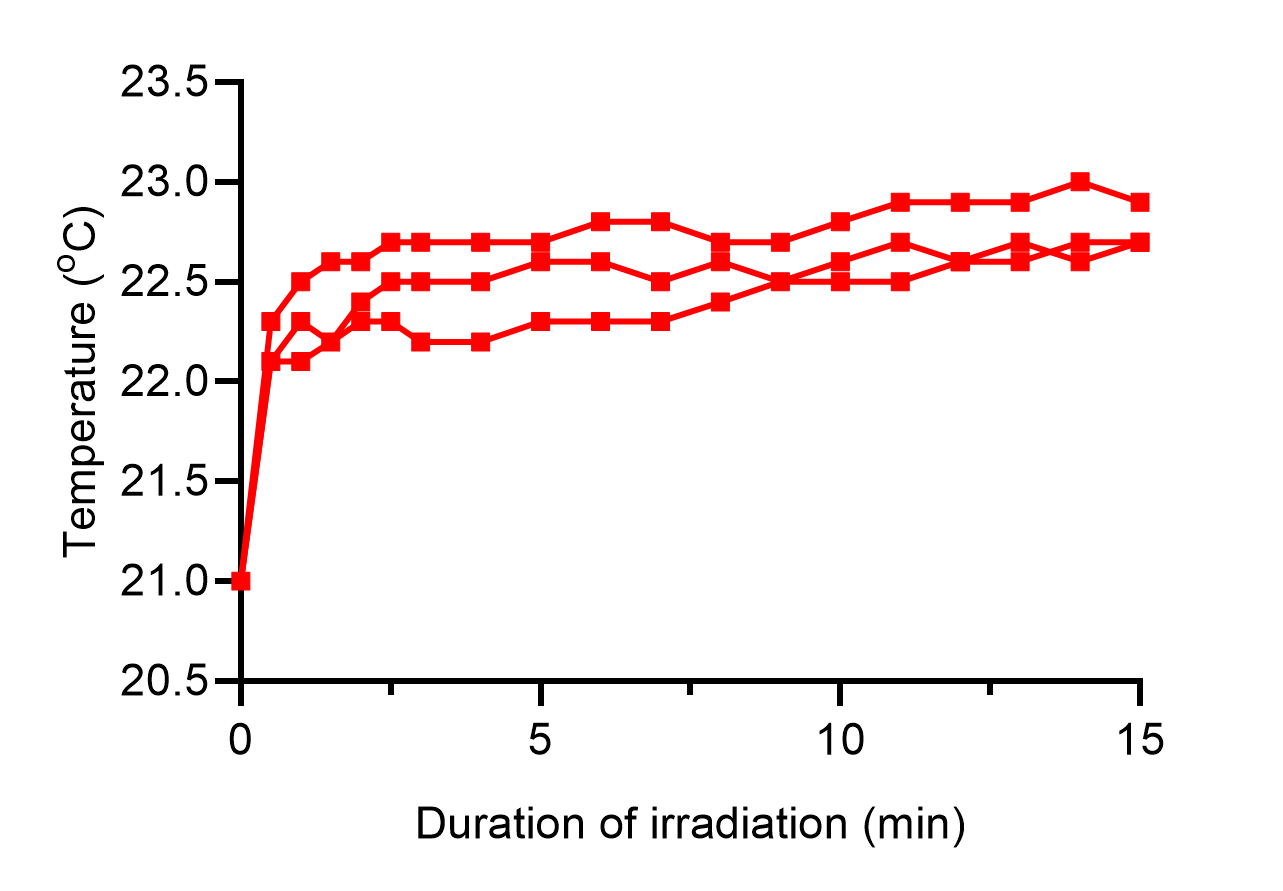


**Supplementary Figure 10.** The temperature profiles of PBS suspension containing Mito(T)-pep-Nuc(T) during the 633-nm laser irradiation (1 W/cm^2^ for 15 min). Three independent tests were conducted and shown individually.


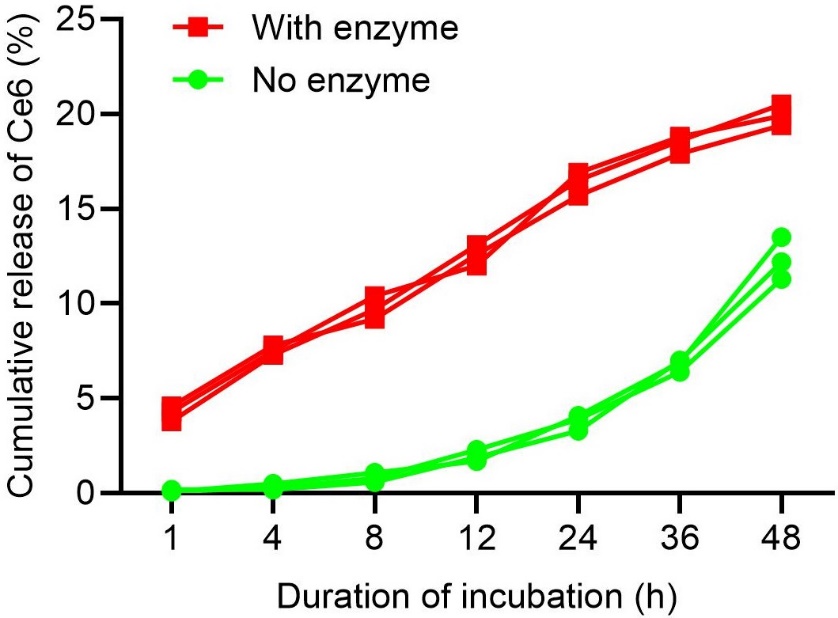


**Supplementary Figure 11.** Cumulative release of Ce6 payload in a course of 48 h. The Mito(T)-pep-Nuc(T) consisted of Ce6 and WONPs at optimized concentrations were used and incubated in PBS suspension supplemented with or without Cathepsin B enzymes (n=3).


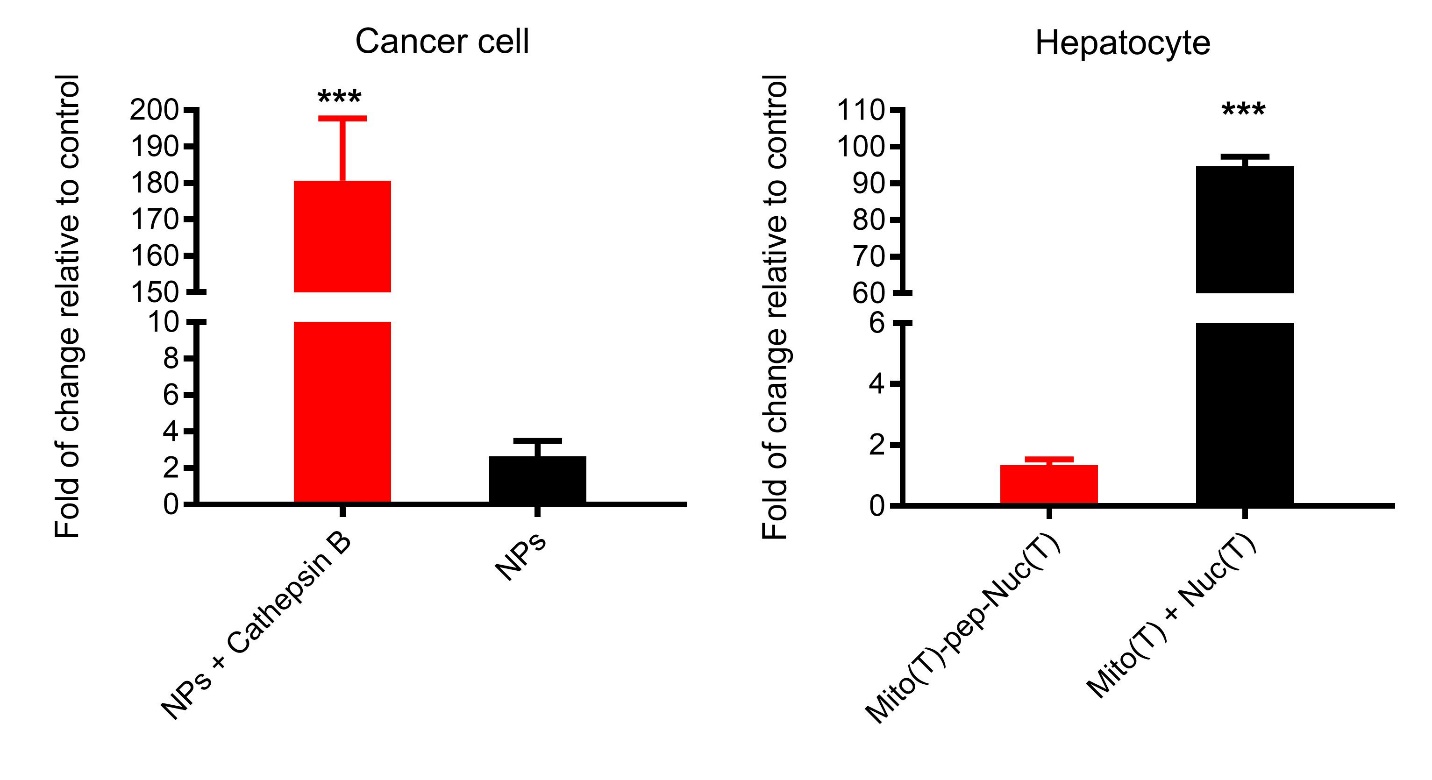


**Supplementary Figure 12.** Changes of the level of intracellular ROS as measured by flow-cytometry shown in **Figure 2c**. These results were presented as fold of change relative to the value of cells in the control group. Data are presented as mean ± s.e.m. (n=3). *P* values were calculated by Student’s *t-*test (****p*<0.001).


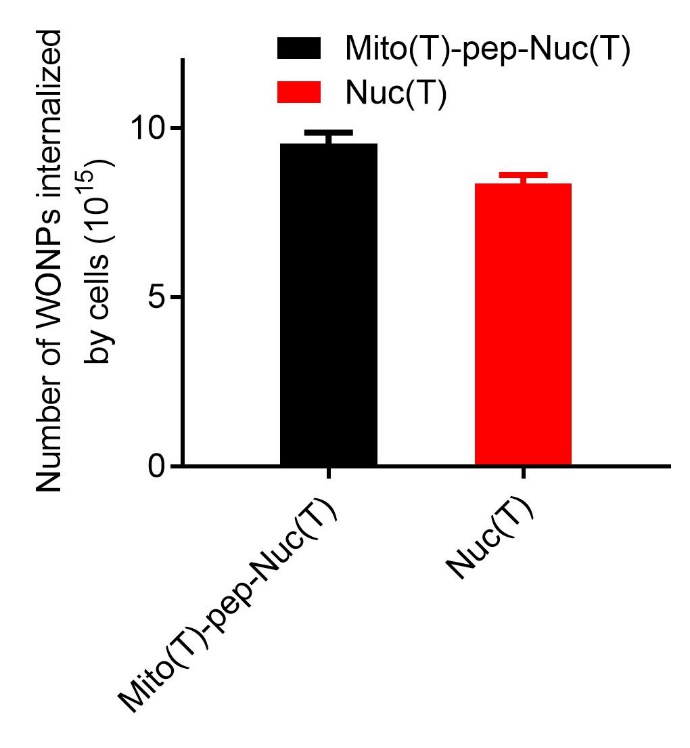


**Supplementary Figure 13.** The number of WONPs internalized by hepatocytes. In this case, the hepatocytes seeded at a density of 1 × 10^5^ cells/well was incubated with 1 mL culture medium containing either Mito(T)-pep-Nuc(T) or Nuc(T) (both at a concentration of 2.35 × 10^16^ particles/mL in terms of WONPs). The content of intracellular tungsten was quantified using ICP-MS at 4 h post incubation. Data are presented as mean ± s.e.m. (n=3). *P* values were calculated by Student’s *t-*test. The difference between these two groups is not statistical significance.


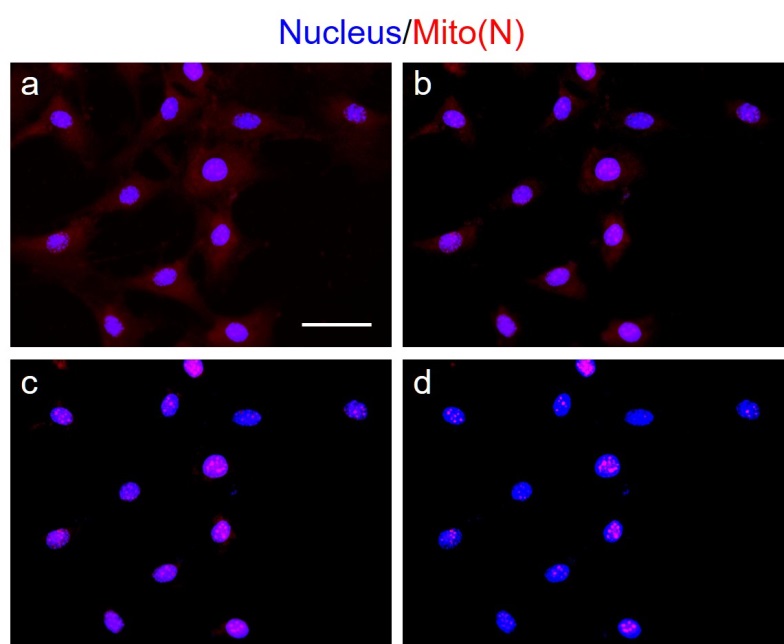


**Supplementary Figure 14.** Time-lapse fluorescence study showing the transient accumulation of Nuc(T) in nuclei, that were labeled in red and blue, respectively. The micrographs were collected after the cells have been incubated with Mito(T)-pep-Nuc(T) for (a) 30 min, (b) 45 min, (c) 60 min, and (d) 90 min. Scale bar: 50 μm.


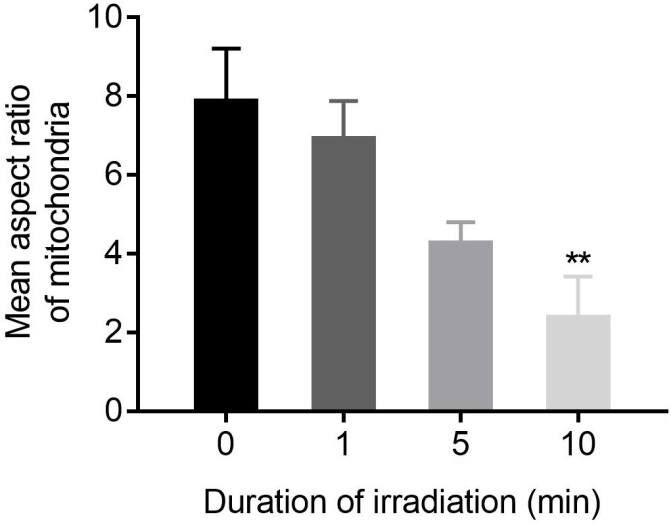


**Supplementary Figure 15.** The mean diameter of mitochondria as a function of the duration of 633-nm laser irradiation. Data are presented as mean ± s.e.m. (n=3). *P* values were calculated by One-Way ANOVA (***p*<0.005).


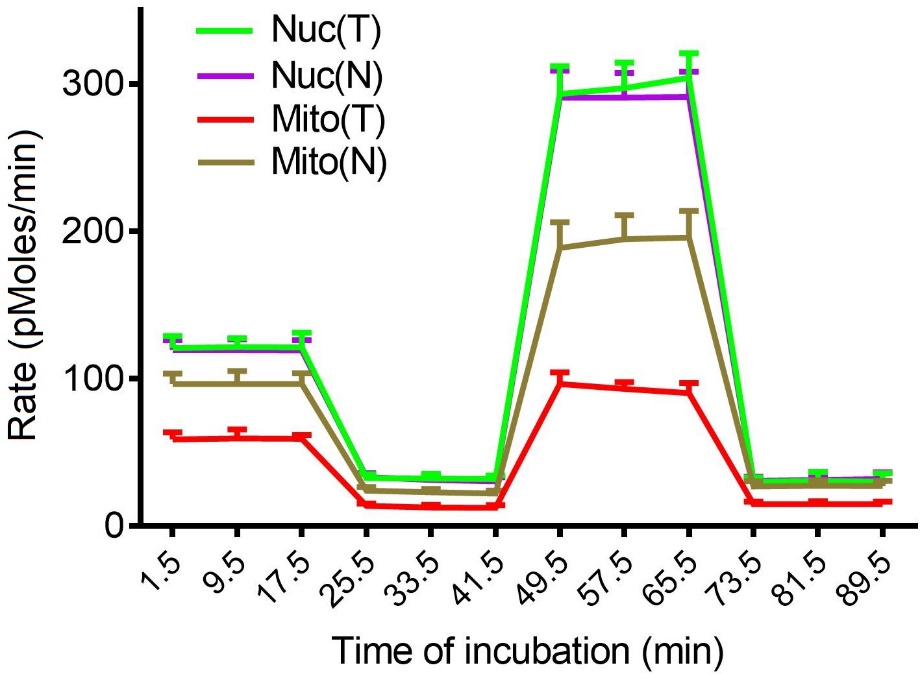


**Supplementary Figure 16.** The OCR profiles of hepatocytes received treatments mediated by Nuc(T), Nuc(N) and Mito(T), Mito(N). ‘T’ and ‘N’ stand for selective and non-selective toward a given type of organelle, respectively. The concentration of either Nuc or Mito was equivalent to that of Mito(T)-pep-Nuc(T) in terms of tungsten or silica, respectively. The sequential laser irradiation was conducted as done for Mito(T)-pep-Nuc(T) regardless of the type of these four therapeutics. Data are presented as mean ± s.e.m. (n=3).


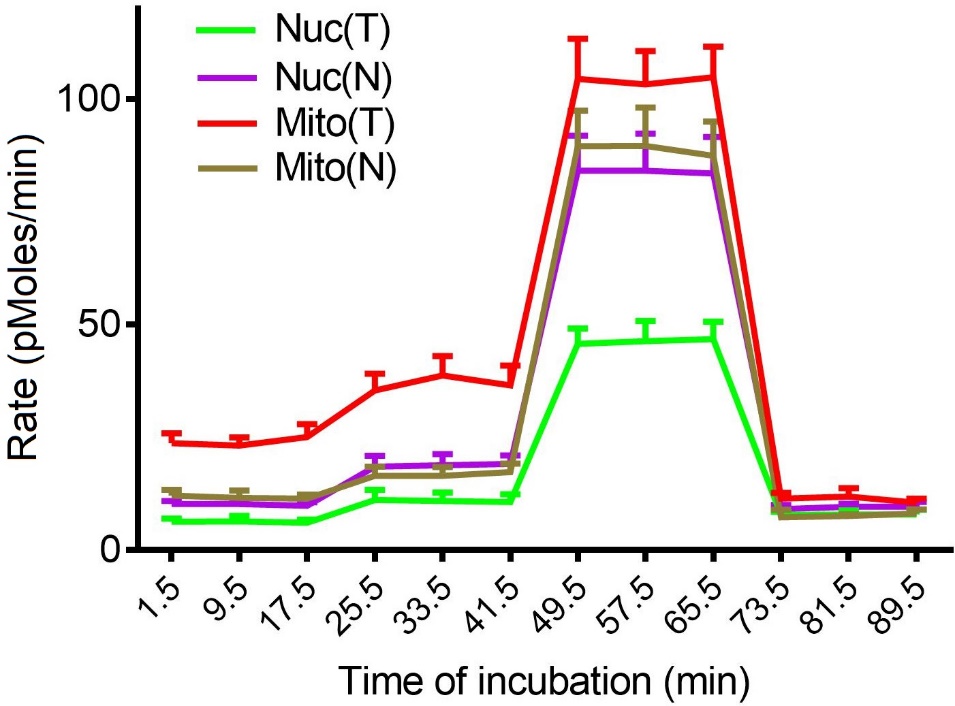


**Supplementary Figure 17.** The ECAR profiles of hepatocytes received treatments mediated by Nuc(T), Nuc(N) Mito(T), and Mito(N). The definitions of therapeutics and the conditions of analysis were the same as those mentioned in the caption of **Supplementary Figure 12**. Data are presented as mean ± s.e.m. (n=3).


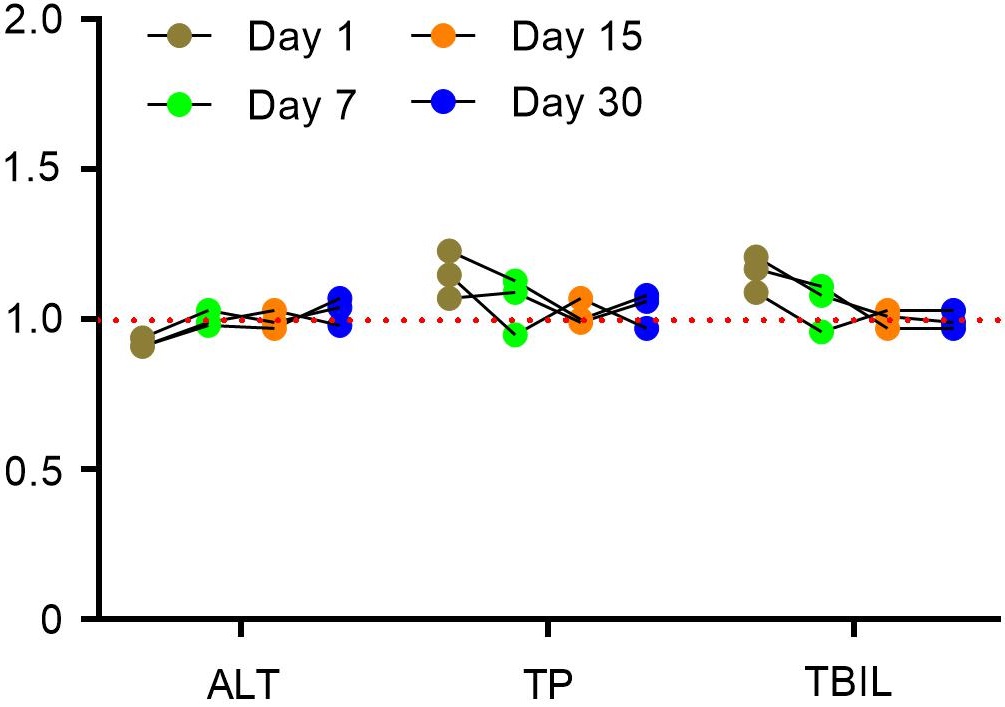


**Supplementary Figure 18**. The changes in liver functions of normal mice received 633-nm laser irradiation (1 W/cm^2^ for 15 min). Three typical biomarkers including ALT (alanine aminotransferase), TP (total protein), TBIL (total bilirubin) were seleced for analyses (n=3). The data were presented as the change in expression of level for an individual mouse to better reflect the abnormality caused by laser exposure.


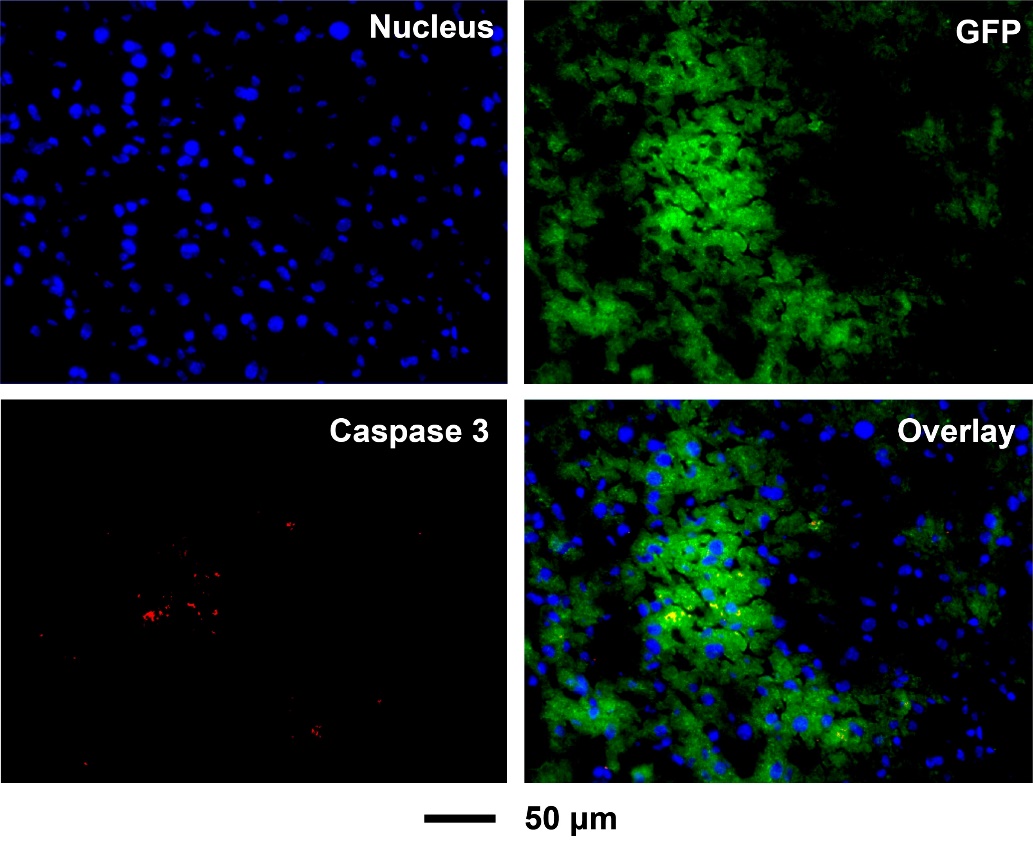


**Supplementary Figure 19.** Immunofluorescence images of liver tissues of mice received Mito(T)-Nuc(T)-mediated treatment. The cell nucleus, expression of Caspase 3 proteins was stained in blue and red with Hoechst 33342 and antibody, respectively. Cancer cells metastasized to liver were recognized according to their expression of GFP proteins (green in color).


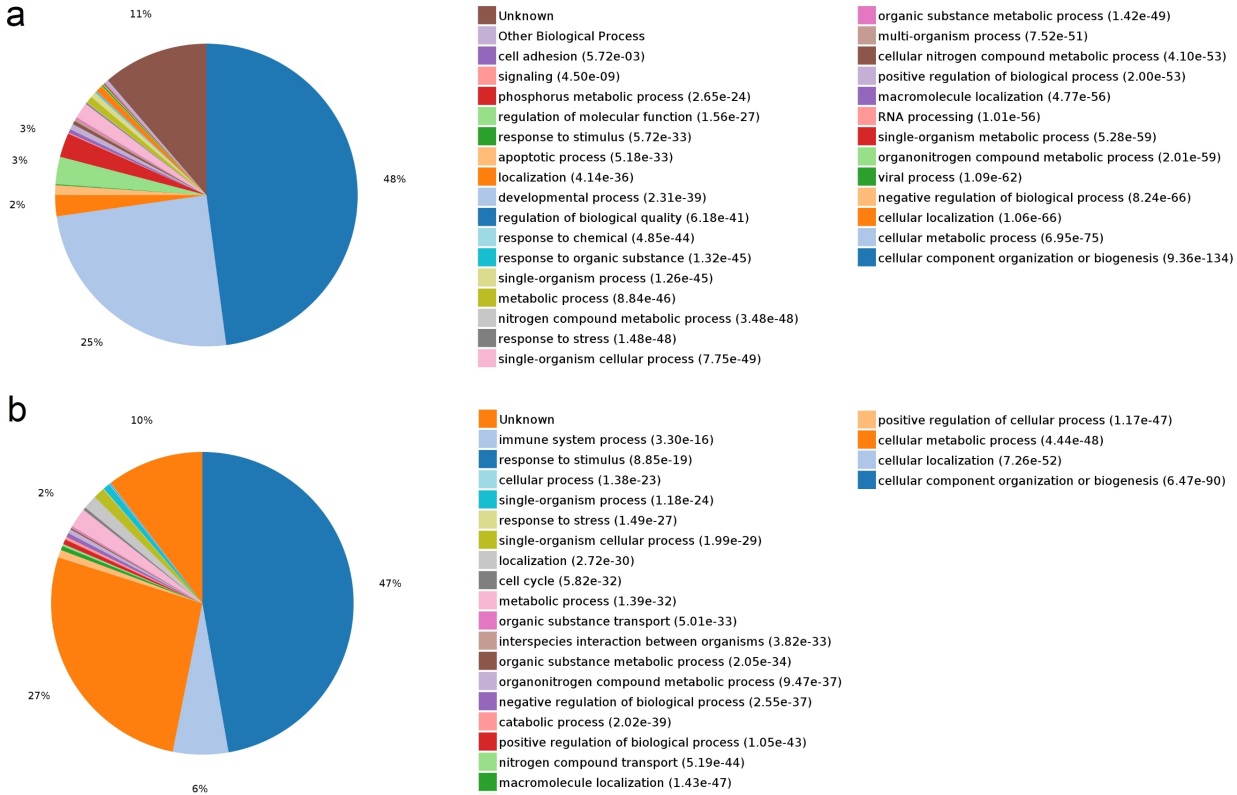


**Supplementary Figure 20.** Expressed (a) up-regulated and (b) down-regulated genes of enriched biological process-associated GO annotations in Mito(T)-pep-Nuc(T) group.


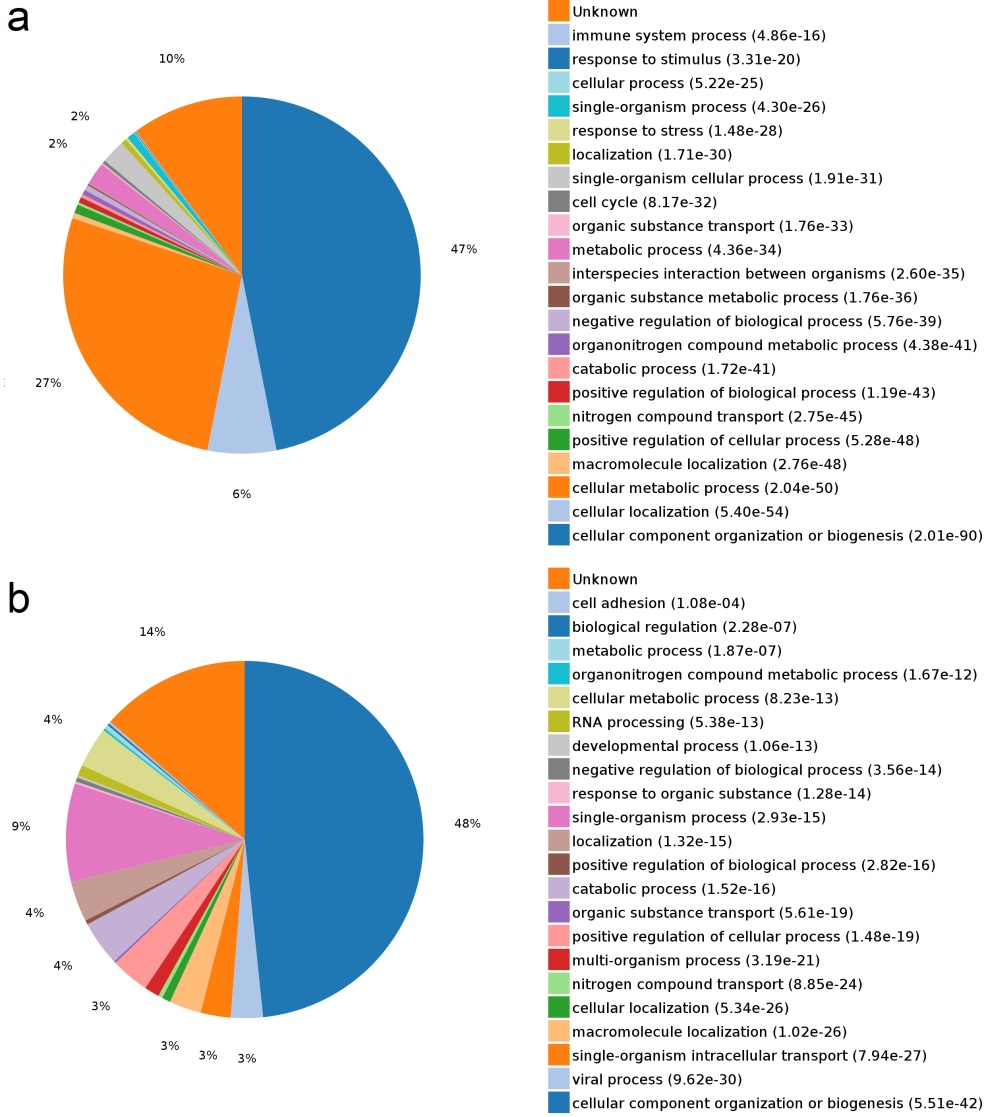


**Supplementary Figure 21.** Expressed (a) up-regulated and (b) down-regulated genes of enriched biological process-associated GO annotations in Mito(T)-pep-Nuc(N) group.


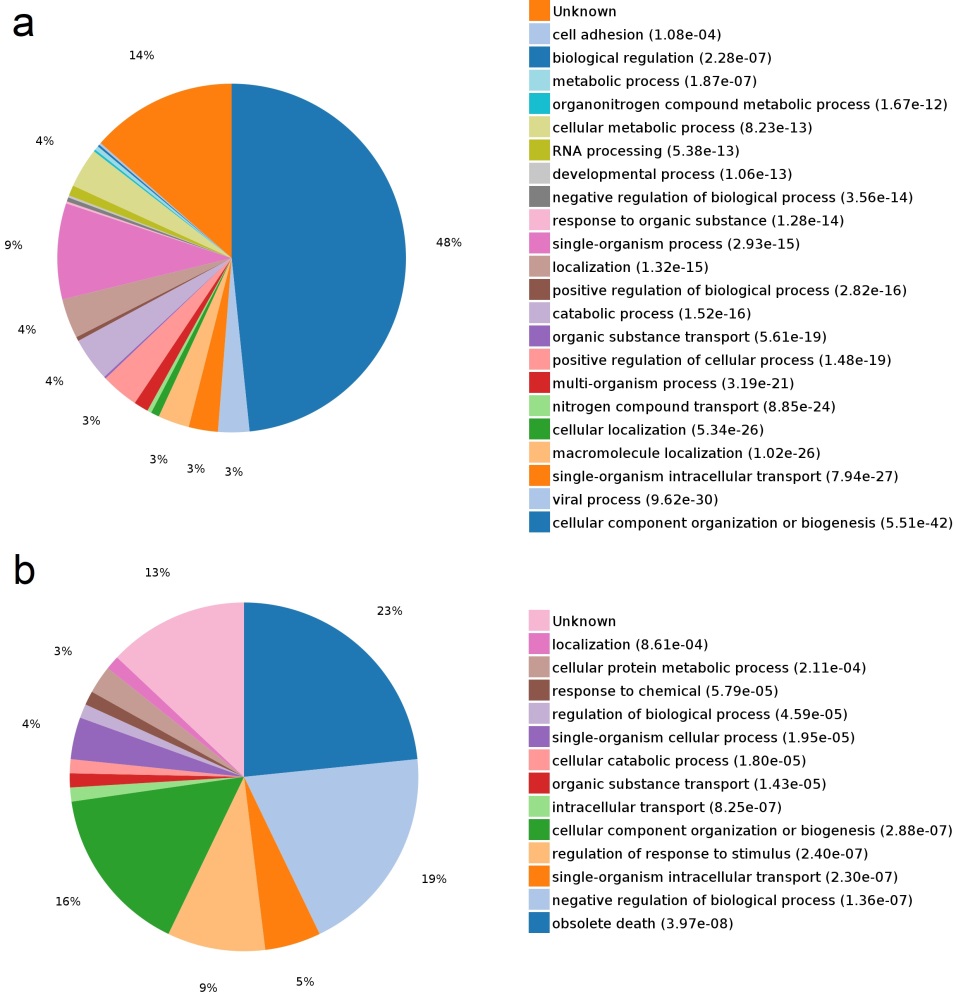


**Supplementary Figure 22.** Expressed (a) up-regulated and (b) down-regulated genes of enriched biological process-associated GO annotations in Mito(N)-pep-Nuc(T) group.


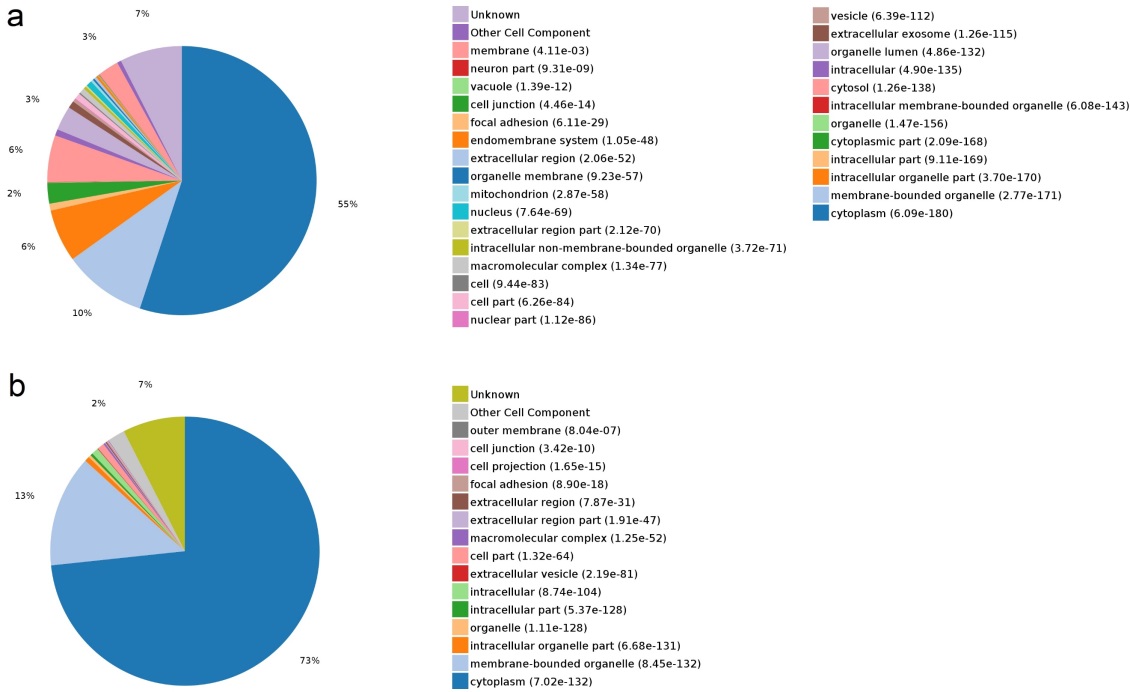


**Supplementary Figure 23.** Expressed (a) up-regulated and (b) down-regulated genes of enriched cell component-associated GO annotations in Mito(T)-pep-Nuc(T) group.


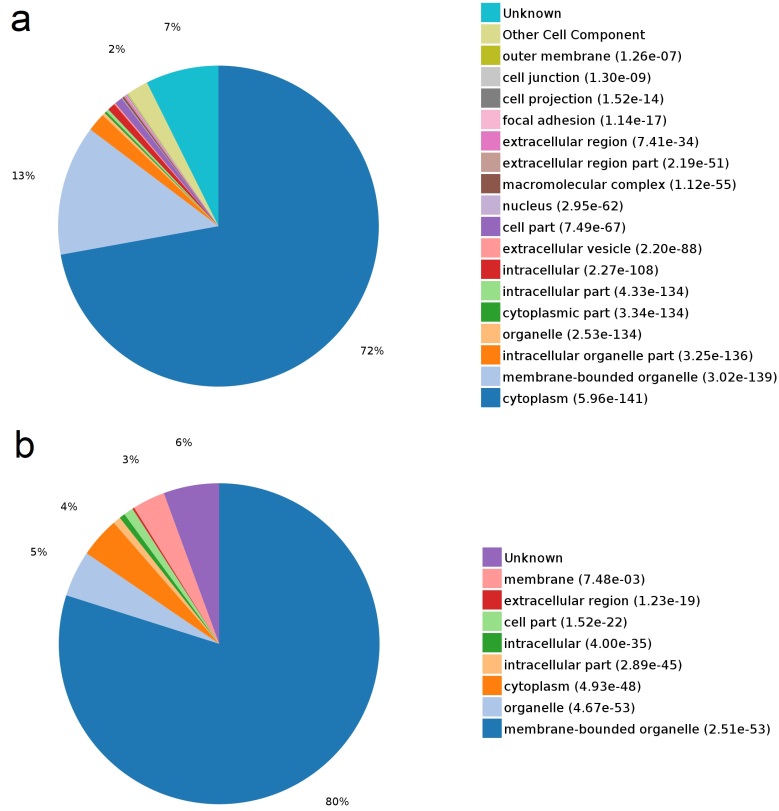


**Supplementary Figure 24.** Expressed (a) up-regulated and (b) down-regulated genes of enriched cell component-associated GO annotations in Mito(T)-pep-Nuc(N) group.


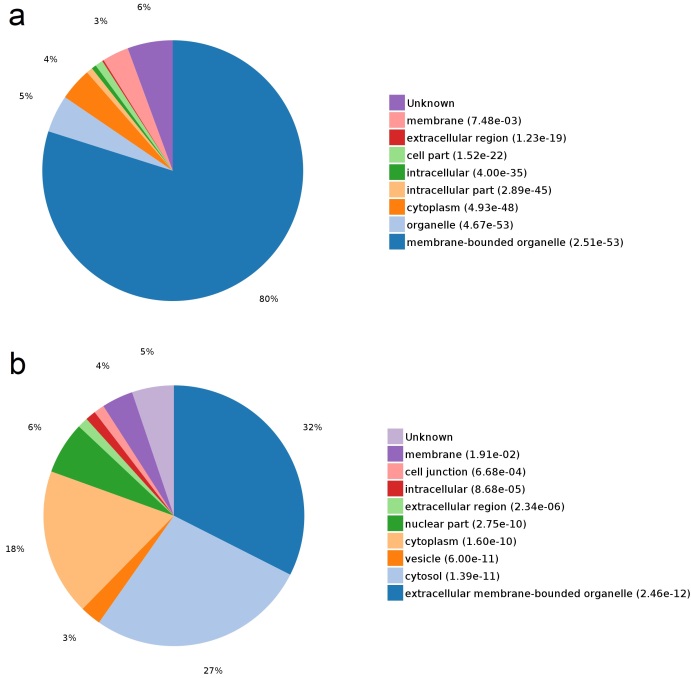


**Supplementary Figure 25.** Expressed (a) up-regulated and (b) down-regulated genes of enriched cell component-associated GO annotations in Mito(N)-pep-Nuc(T) group.


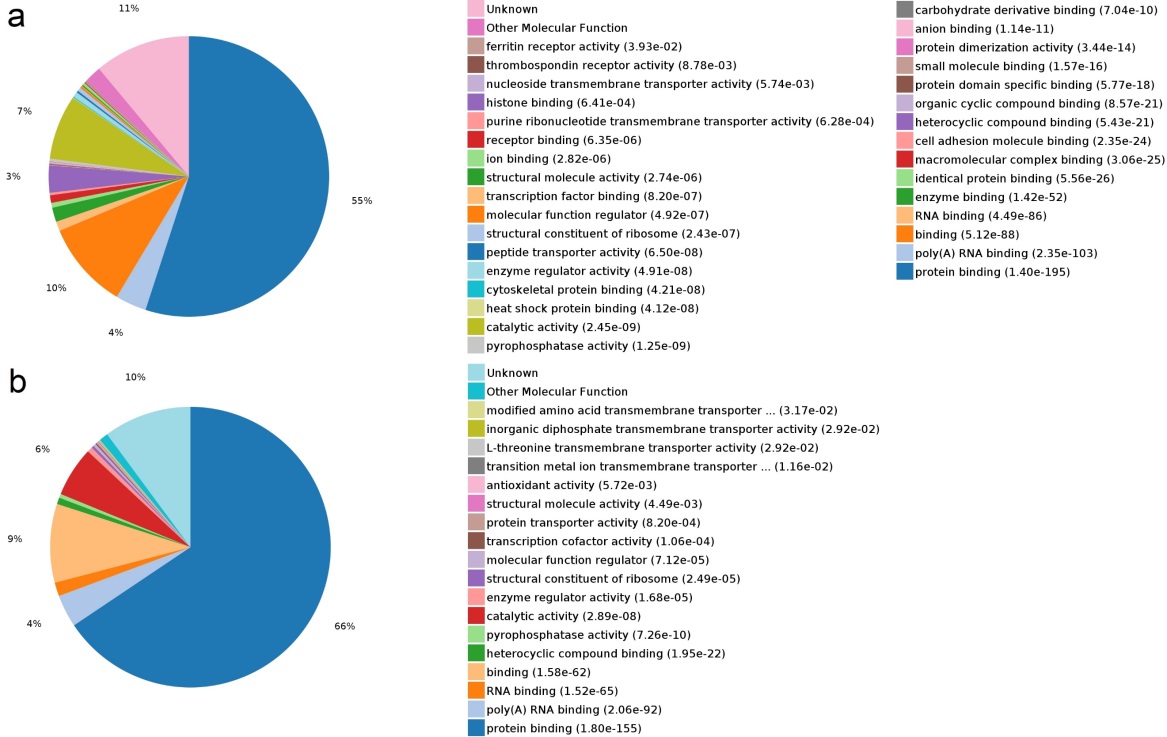


**Supplementary Figure 26.** Expressed (a) up-regulated and (b) down-regulated genes of enriched molecular function-associated GO annotations in Mito(T)-pep-Nuc(T) group.


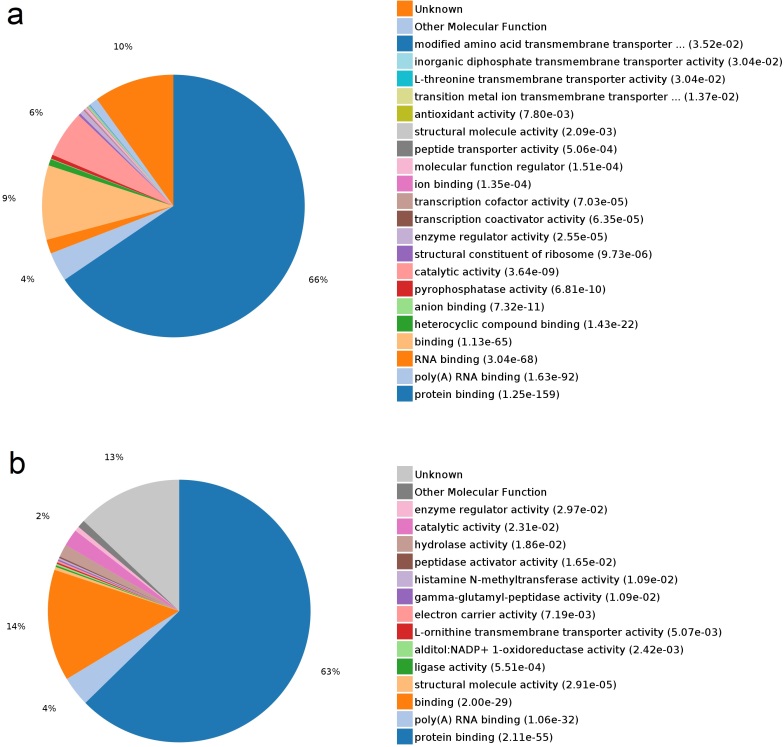


**Supplementary Figure 27.** Expressed (a) up-regulated and (b) down-regulated genes of enriched molecular function-associated GO annotations in Mito(T)-pep-Nuc(N) group.


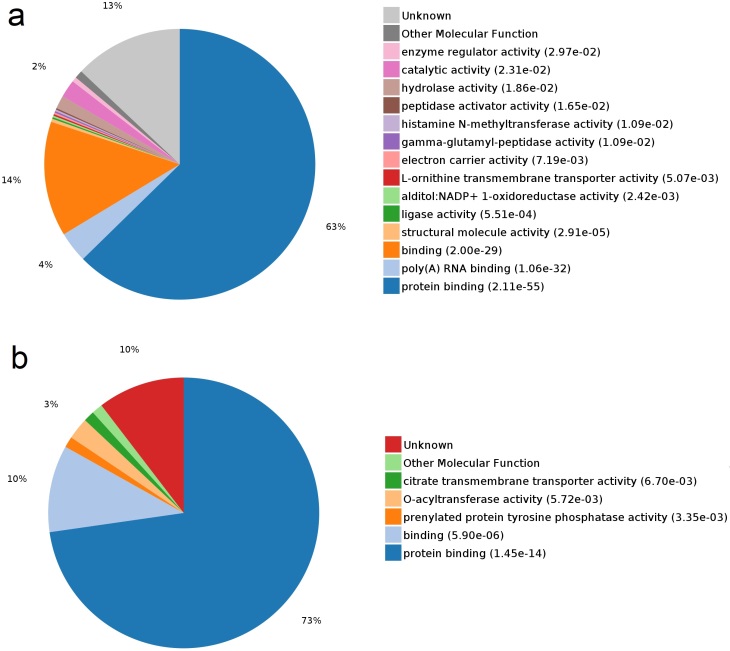


**Supplementary Figure 28.** Expressed (a) up-regulated and (b) down-regulated genes of enriched molecular function-associated GO annotations in Mito(N)-pep-Nuc(T) group.


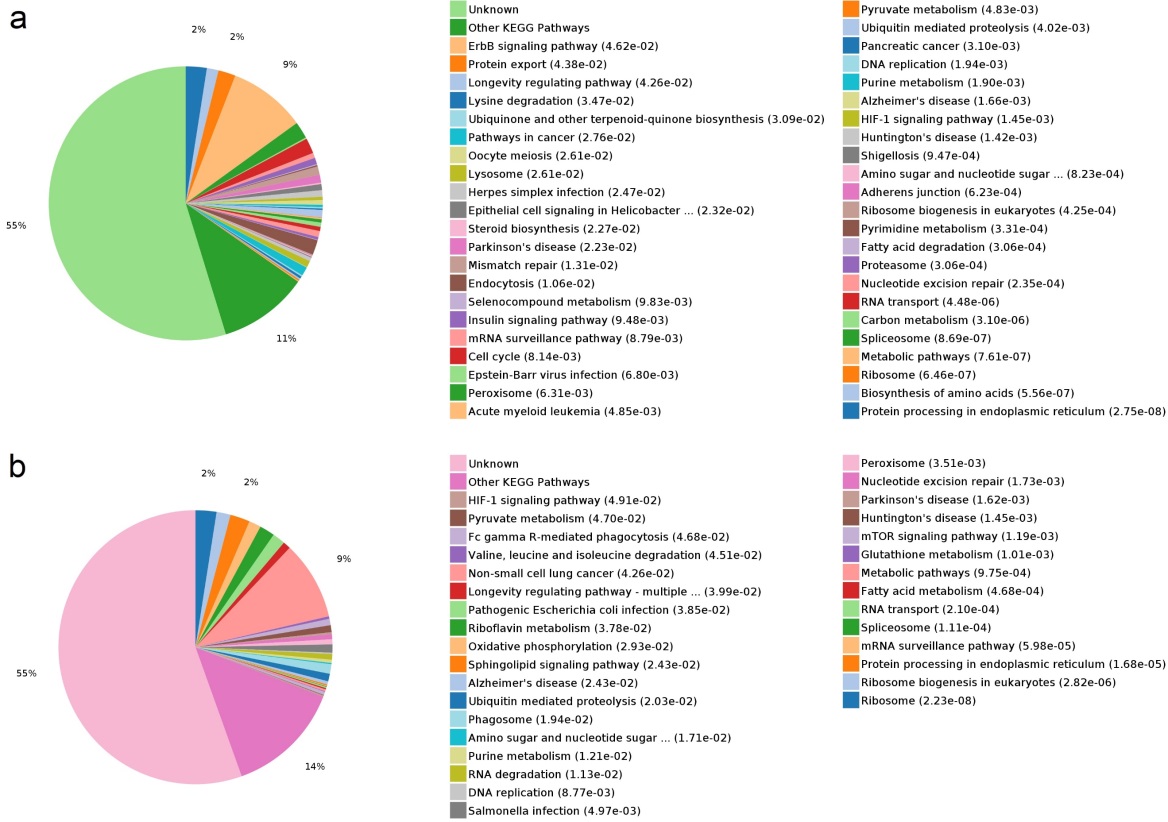


**Supplementary Figure 29.** Expressed (a) up-regulated and (b) down-regulated genes of enriched KEGG pathway-associated GO annotations in Mito(T)-pep-Nuc(T) group.


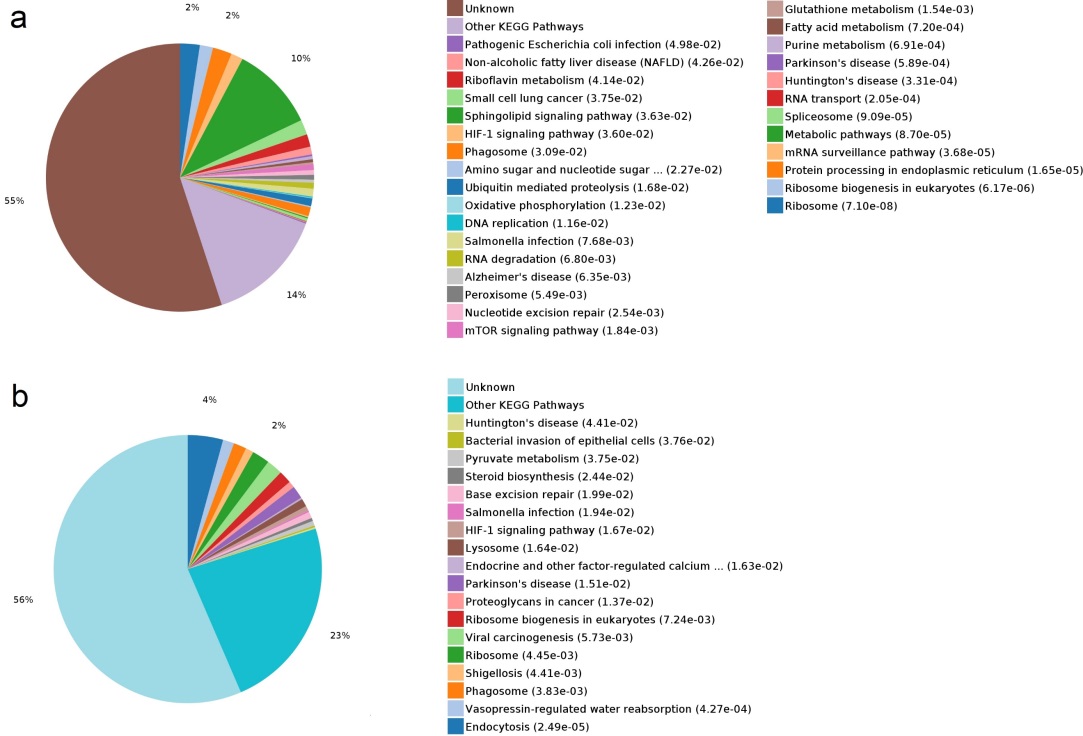


**Supplementary Figure 30.** Expressed (a) up-regulated and (b) down-regulated genes of enriched KEGG pathway-associated GO annotations in Mito(T)-pep-Nuc(N) group.


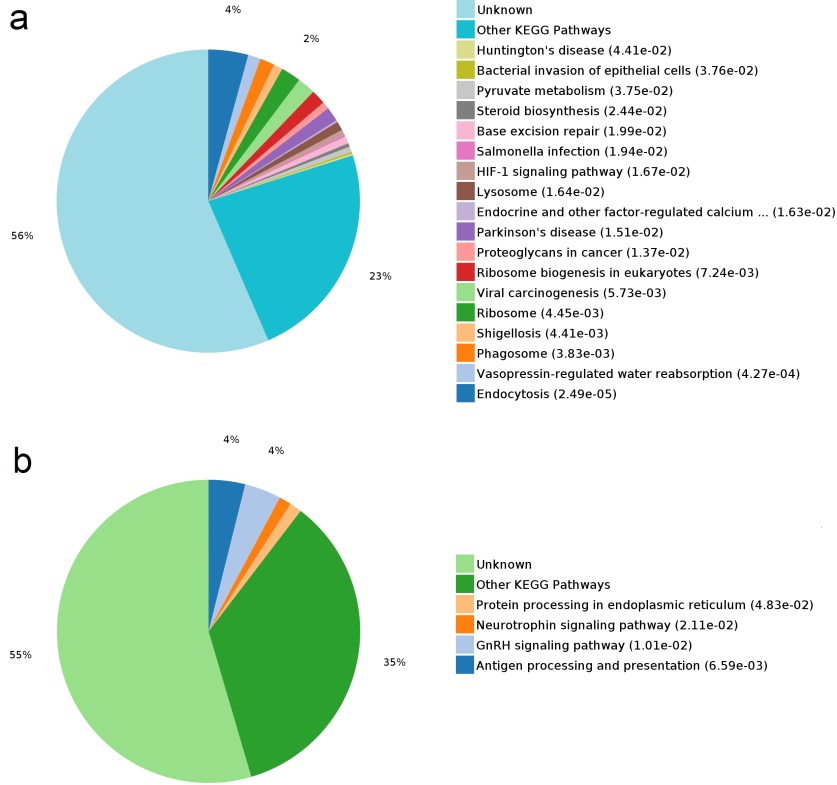


**Supplementary Figure 31.** Expressed (a) up-regulated and (b) down-regulated genes of enriched KEGG pathway-associated GO annotations in Mito(N)-pep-Nuc(T) group.


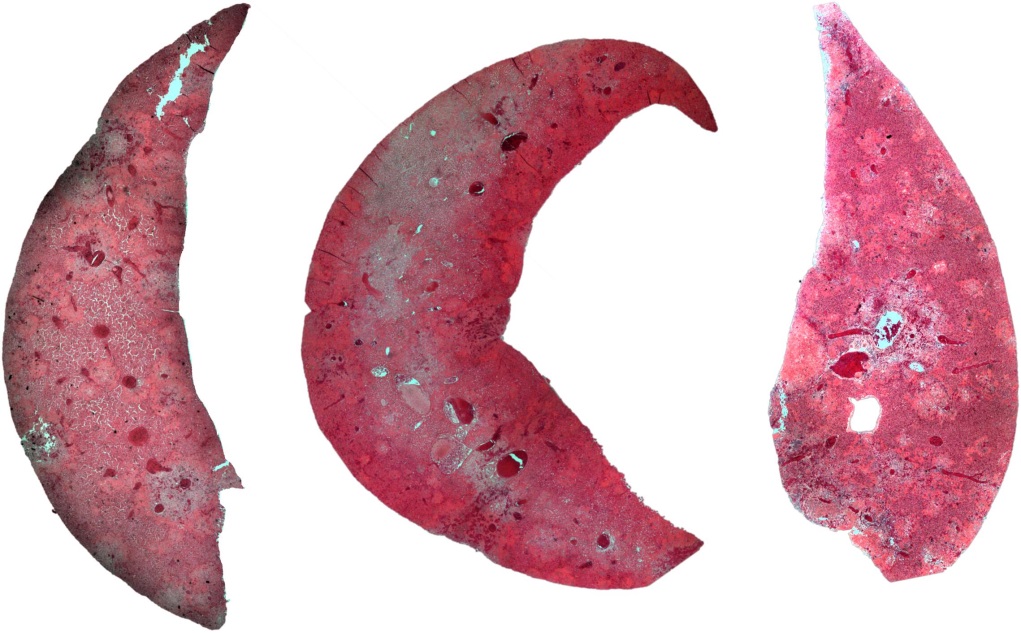


**Supplementary Figure 32.** Necropsy showing the liver tissues of mice in the control group.


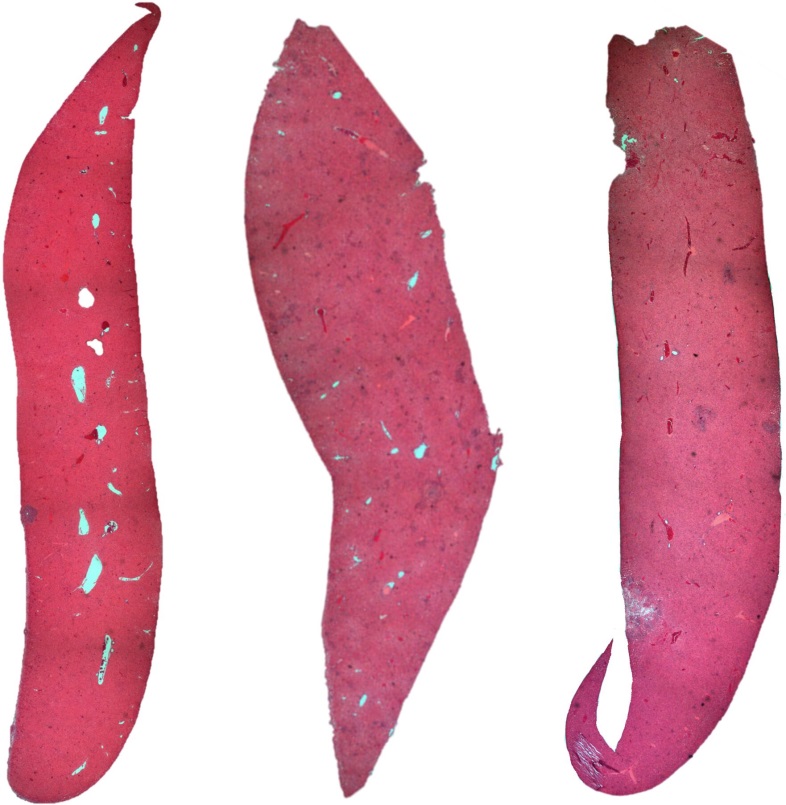


**Supplementary Figure 33.** Necropsy showing the liver tissues of mice in Mito(T)-pep-Nuc(T) group.


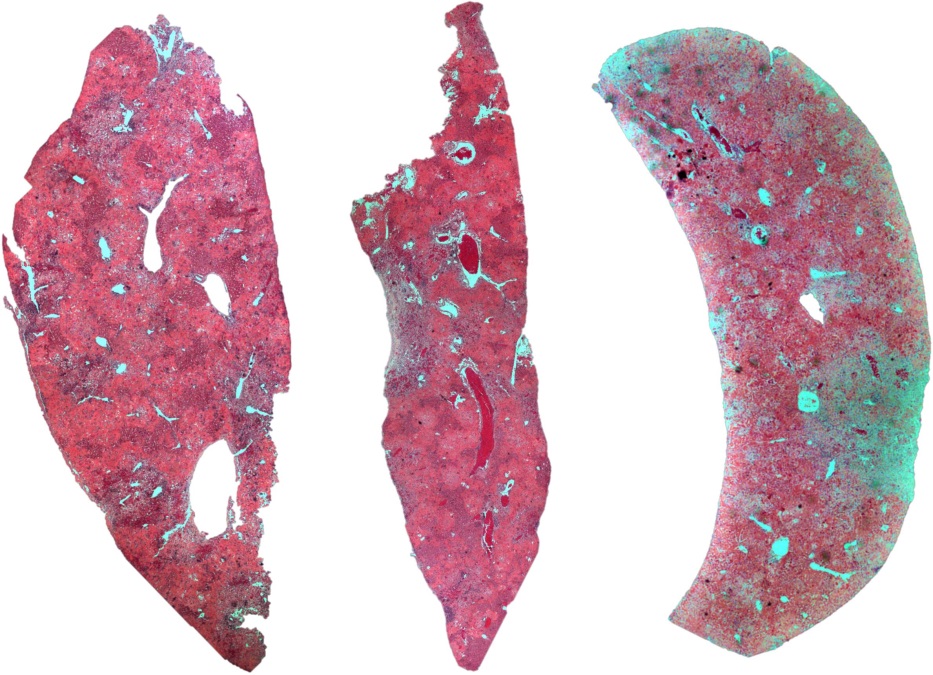


**Supplementary Figure 34.** Necropsy showing the liver tissues of mice in Mito(T)-pep-Nuc(N) group.


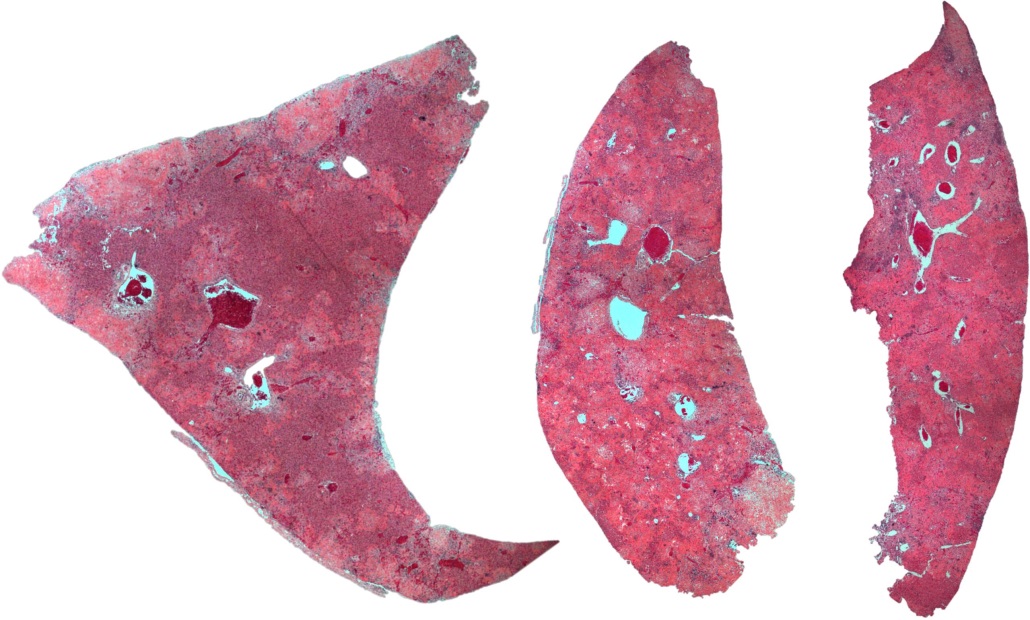


**Supplementary Figure 35.** Necropsy showing the liver tissues of mice in Mito(N)-pep-Nuc(T) group.


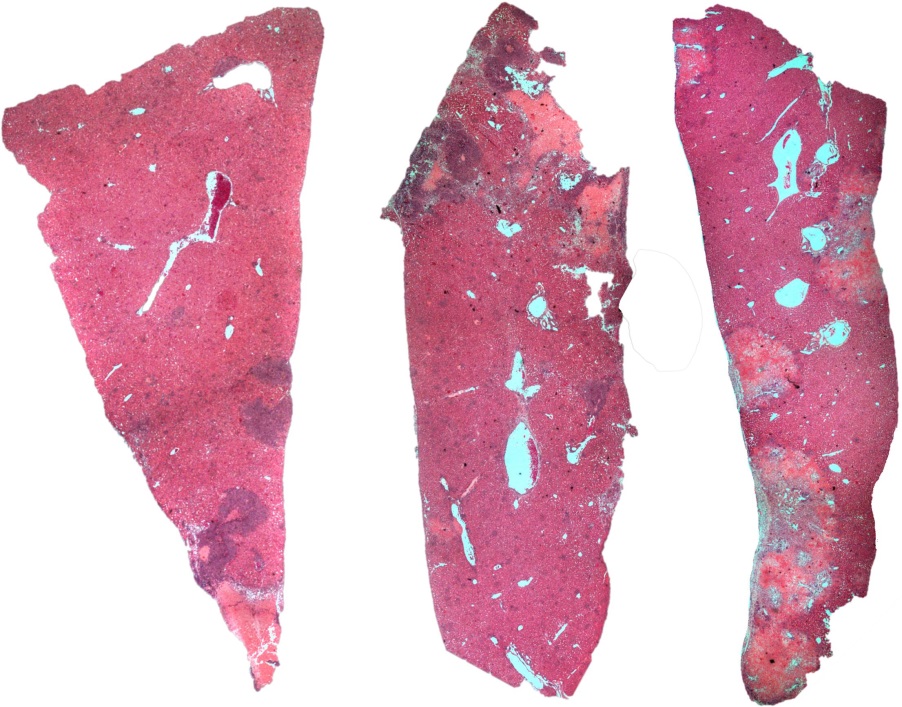


**Supplementary Figure 36.** Necropsy showing the liver tissues of mice received sorafenib.
